# TMED3 Complex Mediates ER Stress‐Associated Secretion of CFTR, Pendrin, and SARS‐CoV‐2 Spike

**DOI:** 10.1002/advs.202105320

**Published:** 2022-06-24

**Authors:** Hak Park, Soo Kyung Seo, Ju‐Ri Sim, Su Jin Hwang, Ye Jin Kim, Dong Hoon Shin, Dong Geon Jang, Shin Hye Noh, Pil‐Gu Park, Si Hwan Ko, Mi Hwa Shin, Jae Young Choi, Yukishige Ito, Chung‐Min Kang, Jae Myun Lee, Min Goo Lee

**Affiliations:** ^1^ Department of Pharmacology Severance Biomedical Science Institute Yonsei University College of Medicine Seoul 03722 Korea; ^2^ Department of Laboratory Medicine Severance Hospital Yonsei University College of Medicine Seoul 03722 Korea; ^3^ Graduate School of Medical Science Brain Korea 21 Project Yonsei University College of Medicine Seoul 03722 Korea; ^4^ Department of Microbiology and Immunology Institute for Immunology and Immunological Diseases Yonsei University College of Medicine Seoul 03722 Korea; ^5^ Department of Otorhinolaryngology Yonsei University College of Medicine Seoul 03722 Korea; ^6^ Cluster for Pioneering Research RIKEN Wako Saitama 351‐0198 Japan; ^7^ Graduate School of Science Osaka University Toyonaka Osaka 560‐0043 Japan; ^8^ Department of Pediatric Dentistry College of Dentistry Yonsei University Seoul 03722 Korea

**Keywords:** CFTR, pendrin, SARS‐CoV‐2 spike, TMED, UPS

## Abstract

Under ER stress conditions, the ER form of transmembrane proteins can reach the plasma membrane via a Golgi‐independent unconventional protein secretion (UPS) pathway. However, the targeting mechanisms of membrane proteins for UPS are unknown. Here, this study reports that TMED proteins play a critical role in the ER stress‐associated UPS of transmembrane proteins. The gene silencing results reveal that TMED2, TMED3, TMED9 and TMED10 are involved in the UPS of transmembrane proteins, such as CFTR, pendrin and SARS‐CoV‐2 Spike. Subsequent mechanistic analyses indicate that TMED3 recognizes the ER core‐glycosylated protein cargos and that the heteromeric TMED2/3/9/10 complex mediates their UPS. Co‐expression of all four TMEDs improves, while each single expression reduces, the UPS and ion transport function of trafficking‐deficient ΔF508‐CFTR and p.H723R‐pendrin, which cause cystic fibrosis and Pendred syndrome, respectively. In contrast, TMED2/3/9/10 silencing reduces SARS‐CoV‐2 viral release. These results provide evidence for a common role of TMED3 and related TMEDs in the ER stress‐associated, Golgi‐independent secretion of transmembrane proteins.

## Graphical abstract

This study highlights the critical role of the TMED3 complex in the Golgi‐independent unconventional protein secretion (UPS) of transmembrane proteins, such as ΔF508‐CFTR, p.H723R‐pendrin and SARS‐CoV‐2 Spike. Under ER stress conditions, TMED3 initially recognizes the ER‐accumulated membrane proteins and the TMED2/3/9/10 heteromeric complex facilitates the UPS of these membrane cargos.

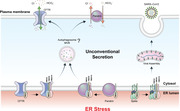

## 1 Introduction

Most secretory and membrane proteins are exported from the endoplasmic reticulum (ER) and trafficked to the Golgi en route to their final destinations. In addition to this conventional secretory pathway, recent discoveries have increasingly revealed transmembrane proteins using alternative secretory pathways that bypass the ER‐to‐Golgi route, which are collectively referred to as unconventional protein secretion (UPS) pathways.^[^ ^1^, ^2^, ^3^ ^]^ In particular, under blocked ER‐to‐Golgi transport or ER stress conditions, misfolded cystic fibrosis transmembrane conductance regulator (CFTR) and pendrin proteins, which are responsible for cystic fibrosis (CF) and specialized forms of congenital hearing loss (Pendred syndrome and DFNB4), respectively, can reach the plasma membrane via a Golgi‐independent UPS route.^[^ ^4^, ^5^ ^]^ Although several factors such as Golgi reassembly‐stacking proteins (GRASPs) and inositol‐requiring enzyme 1α (IRE1α) kinase cascades have been shown to be involved,^[^ ^4^, ^6^ ^]^ the molecular mechanisms underlying ER stress‐associated protein secretion, including the sorting determinants for how UPS substrates are selected, remain largely unknown.

CFTR is a cyclic AMP (cAMP) regulated anion channel conducting Cl^−^ and HCO_3_^−^ at the apical membrane of epithelial cells.^[^ ^7^ ^]^ As a glycoprotein, CFTR is initially core‐glycosylated (band B) in the ER by two asparagine (N)‐linked oligosaccharides, which mediate interactions with ER lectin‐like proteins such as calnexin for ER folding cycles.^[^ ^8^ ^]^ Correctly folded CFTR proteins traverse to the Golgi, undergo complex‐glycan modification (band C), and are ultimately directed to the apical surface of epithelial tissues.^[^ ^9^ ^]^ The most common mutation in CF, deletion of the phenylalanine residue at position 508 (ΔF508), results in protein misfolding and subsequent degradation via the ER quality control (ERQC) and ER‐associated degradation (ERAD) pathways.^[^ ^10^ ^]^ Consequently, ΔF508‐CFTR proteins remain in the core‐glycosylated form within the ER with negligible quantities expressed at the plasma membrane. Pendrin is a transmembrane protein that transports anions such as Cl^−^, I^−^, and HCO_3_^−^ in the inner ear and thyroid follicles.^[^ ^11^ ^]^ The most frequent disease‐causing mutation of pendrin, p.H723R (His723Arg) results in protein misfolding, ER retention, and degradation via ERAD, being similar to the fate of ΔF508‐CFTR.^[^ ^12^ ^]^ Although defective in protein folding, ΔF508‐CFTR and p.H723R‐pendrin proteins retain some functional activity once expressed on the cell surface. Notably, upregulation of UPS pathways resulting from GRASP55 overexpression or IRE1*α* kinase activation has been shown to directly induce the cell surface expression of the ER form of core‐glycosylated CFTR and pendrin, bypassing the Golgi, suggesting that UPS activation could be a strategy to treat diseases caused by trafficking‐defective CFTR and pendrin.^[^ ^4^, ^6^ ^]^

Severe acute respiratory syndrome coronavirus‐2 (SARS‐CoV‐2) is the etiologic agent of COVID‐19 and belongs to the genus *β*‐coronavirus (group 2).^[^ ^13^ ^]^ The spike (S) protein is an N‐glycosylated transmembrane protein and class I fusion protein in the viral envelope, which plays a critical role in the infection of host cells. The coronavirus S protein comprises two functional subunits, S1 and S2. The former contains the receptor‐binding domain that binds to the host cell receptor angiotensin‐converting enzyme 2 (ACE2), whilst the latter retains the fusion peptide that is responsible for the fusion between the viral envelope and host cell membrane.^[^ ^13^ ^]^ This protein also serves an important role in the humoral immune response in the host and hence is the principal focus of vaccine development.^[^ ^14^ ^]^ Subsequent to synthesis in the ER, the *β*‐coronavirus S proteins are transported to the viral assembly sites and the plasma membrane of host cells.^[^ ^13^, ^15^ ^]^ SARS‐CoV‐2 viral infections and related cellular stresses are known to evoke reorganization of the ER and cellular secretory organelles, which results in the viral infection‐induced alterations of secretome profiles.^[^ ^16^, ^17^ ^]^ However, the secretory mechanisms via which S proteins exit the ER and travel to the plasma membrane have not been fully established yet.

The transmembrane emp24 domain‐containing proteins (TMED, also known as p24 proteins) are a family of type I membrane proteins distributed in the membranes of the early secretory pathway. Although TMED proteins have been suggested to function as cargo receptors for the anterograde transport of some secretory cargos^[^ ^18^ ^]^ or as primary receptors for the small GTPase of coat protein complex I (COPI)‐vesicle formation,^[^ ^19^ ^]^ most of their functions remain elusive. In the present study, we demonstrate that TMED proteins act as cargo‐recruiting factors for ER stress‐associated protein secretion. More specifically, TMED3 recognizes the ER core‐glycosylated membrane protein cargos, and the TMED2/3/9/10 complex efficiently directs them to the cell surface. This newly discovered role of TMED provides greater mechanistic insight into how ER cargo proteins are selected for UPS during ER stress, and offers new therapeutic strategies for human diseases stemming from trafficking defects and pathogenic expressions of membrane proteins.

## 2 Results

### 2.1 TMED2, TMED3, TMED9, and TMED10 Are Involved in the Unconventional Protein Secretion of CFTR and Pendrin

We initially investigated the role of individual TMED proteins in CFTR UPS. The cell surface expression of folding‐defective ΔF508‐CFTR was induced by the dominant‐inhibitory form of ADP‐ribosylation factor 1 (ARF1‐Q71L), which blocks ER‐to‐Golgi transport.^[^ ^20^ ^]^ In a control experiment for this study, ARF1‐Q71L evoked the cell surface expression of core‐glycosylated wild‐type (WT) and ΔF508 CFTRs but not the non‐UPS cargo of the transferrin receptor (Figure S1A,B, Supporting Information). The ER‐to‐Golgi transport blockade by ARF1‐Q71L induces protein accumulation in the ER and activates the ER stress sensing molecule IRE1*α* kinase.^[^ ^4^ ^]^ Treatment with the IRE1*α* kinase activator, (E)‐2‐(2‐chlorostyryl)‐3,5,6‐trimethyl‐pyrazine (CSTMP), induces the UPS of some core‐glycosylated membrane proteins such as ΔF508‐CFTR and p.H723R‐pendrin.^[^ ^6^ ^]^ It is of note that treatment with CSTMP induced the cell surface expression of ΔF508‐CFTR in CFF‐16HBEge CFTR F508del cells, a bronchial epithelial cell line harboring the ΔF508 CFTR mutation,^[^ ^21^ ^]^ further demonstrating that CFTR is a UPS substrate (Figure S1C,D, Supporting Information).

**Figure 1:**
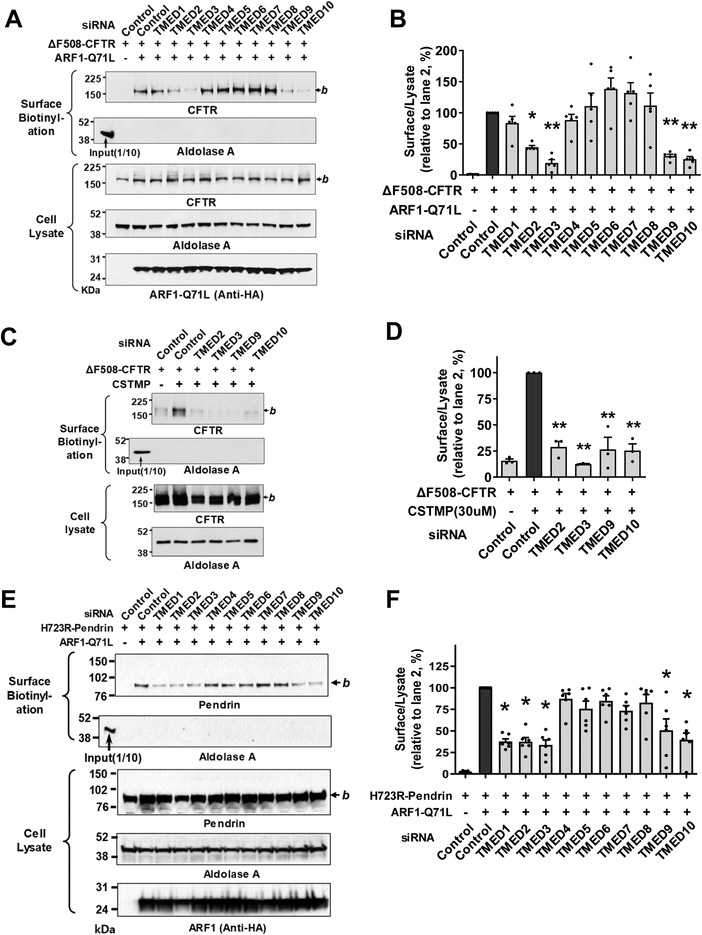
TMED2, 3, 9, 10 are involved in the unconventional secretion of CFTR and pendrin. A,B) Effects of individual *TMED* gene silencing on the unconventional protein secretion (UPS) of ΔF508‐CFTR. The cell surface biotinylation assay was performed in HEK293 cells transfected with control (scrambled) or *TMED‐*specific siRNAs (100 nm each, 48 h) together with plasmids encoding ΔF508‐CFTR. ARF1‐Q71L was co‐expressed in some cells to induce UPS. Representative blots of surface biotinylation assays are shown in (A). The HA‐tagged ARF1‐Q71L was blotted with anti‐HA antibodies. Quantifications of multiple experiments are summarized in (B) (*n* = 5). C,D) Effects of *TMED* gene silencing were analyzed in HEK293 cells treated with the IRE1*α* kinase activator, (E)‐2‐(2‐chlorostyryl)‐3,5,6‐trimethyl‐pyrazine (CSTMP), to induce UPS. Representative blots are shown in (C) and the results of multiple experiments are summarized in (D) (*n* = 3). E,F) Effects of *TMED* gene silencing on the UPS of p.H723R‐pendrin. The cell surface biotinylation assay was performed in PANC‐1 cells transfected with plasmids encoding p.H723R‐pendrin. ARF1‐Q71L was co‐expressed in some cells to induce UPS. Representative blots of surface biotinylation assays are shown in (E). Quantifications of multiple experiments are summarized in (F) (*n* = 6). Bar graph data are shown as mean ± SEM. *p < 0.05, **p < 0.01: difference from lane 2. Data were analyzed using one‐way analysis of variance, followed by Tukey's multiple comparison test. *b*, core‐glycosylated CFTR, and pendrin.

Analyses of the ten‐membered *TMED* gene knockdown revealed that the silencing of *TMED2, TMED3, TMED9*, and *TMED10* substantially inhibited the ARF1‐Q71L‐induced UPS of ΔF508‐CFTR in HEK293 cells, with *TMED3* silencing being the most effective (**Figure** **1**A,B). The silencing of *TMED2*, *3*, *9*, and *10* also strongly inhibited the CSTMP‐induced UPS of ΔF508‐CFTR (Figure 1C,D). mRNA quantification confirmed the silencing of each *TMED* gene (Figure S2A, Supporting Information). The bronchial respiratory epithelial cells abundantly express the above UPS‐related TMED proteins and the UPS molecular players, such as IRE1*α* and GRASP55 (Table S1, Supporting Information, the Human Protein Atlas), and the expressions of TMED3, IRE1*α* and GRASP55 were confirmed in the model airway epithelia of primarily cultured human nasal epithelial cells (Figure S2B, Supporting Information). Interestingly, the conventional cell surface transport of complex‐glycosylated WT‐CFTR was unaffected by the knockdown of TMED subfamilies (Figure S2C,D, Supporting Information).

Because the folding defective, core‐glycosylated p.H723R‐pendrin also undergoes the ER stress‐induced UPS pathway,^[^ ^5^ ^]^ we next explored whether TMEDs are also involved in pendrin UPS. PANC‐1 cells derived from pancreatic ducts were used to examine the cell surface expression of pendrin.^[^ ^5^ ^]^ Treatments with siRNAs revealed that silencing of similar *TMEDs*, in particular *TMED2*, *3*, *9*, and *10*, strongly inhibited the ARF1‐Q71L‐induced UPS of p.H723R‐pendrin (Figure 1E,F). However, the conventional cell surface transport of WT‐pendrin was unaffected by knockdown of *TMEDs* (Figure S2E,F, Supporting Information), being similar to the case of WT‐CFTR.

### 2.2 TMED3 Is a Central Regulator of Membrane Protein UPS via Bridging Cargo Proteins to other TMEDs

**Figure 2:**
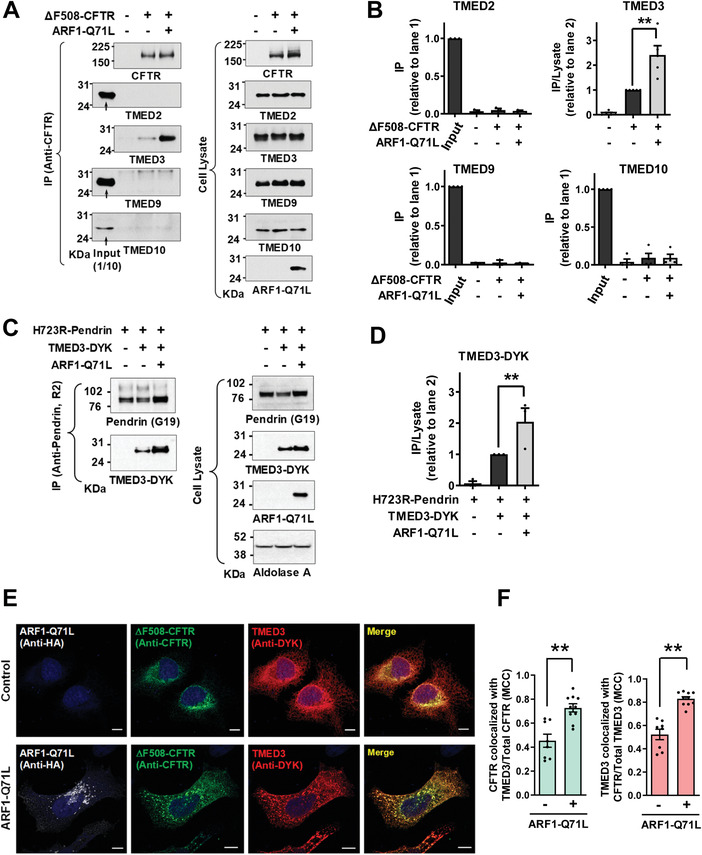
TMED3 associates with CFTR and pendrin. A,B) Co‐immunoprecipitation (co‐IP) experiments between CFTR and TMED proteins (TMED2, TMED3, TMED9, and TMED10) were performed in HEK293 cells expressing ΔF508‐CFTR. Protein samples were precipitated with anti‐CFTR antibodies (M3A7) and blotted with antibodies against each TMED protein. Representative IP blots are shown in (A). Quantifications of multiple experiments are summarized in (B) (*n* = 3–5). CFTR associates with TMED3 but not with other TMEDs. C,D) Co‐IP experiments between pendrin and TMED3 were performed in PANC‐1 cells expressing p.H723R‐pendrin. Protein samples were precipitated with anti‐pendrin antibodies (R2) and blotted with antibodies against TMED3. Representative IP blots are shown in (C). Quantifications of multiple experiments are summarized in (D) (*n* = 3). Co‐IP results with TMED2, TMED9, and TMED10 are presented in Figure S3, Supporting Information. Pendrin associates with TMED3 but not with other TMEDs. E,F) Cellular localizations of TMED3 and ΔF508‐CFTR were analyzed using immunocytochemistry in HeLa cells with DYK‐ TMED3 expressions. CFTR was stained with anti‐CFTR and green fluorophore‐tagged antibodies and DYK‐TMED3 with anti‐DYK and red fluorophore‐tagged antibodies after permeabilization. Representative immunofluorescence images are shown in (E). Quantification of colocalization between TMED3 and CFTR using the Manders’ colocalization coefficient (MCC) is summarized in (F) (*n* = 8–10). Scale bar: 10 µm. Bar graph data are shown as mean ± SEM. **P < 0.01. Data were analyzed using one‐way analysis of variance, followed by Tukey's multiple comparison test (B,D) or a two‐tailed Student's *t*‐test (F).

To identify the mechanism underlying TMED‐mediated regulation of CFTR UPS, the relationships between TMED proteins and CFTR were further examined using co‐immunoprecipitation analyses. When protein samples were precipitated with antibodies against CFTR, only TMED3 (i.e., and not TMED2, TMED9 or TMED10) was detectable in the complex (**Figure** **2**A,B). The protein–protein interaction between ΔF508‐CFTR and TMED3 was minimal under normal conditions. Notably, the blockade of ER‐to‐Golgi transport by ARF1‐Q71L increased the association between ΔF508‐CFTR and TMED3 (Figure 2A,B). Similarly to the case of CFTR, TMED3, but not other TMEDs, directly interacted with p.H723R‐pendrin. In addition, ARF1‐Q71L augmented the interaction between TMED3 and p.H723R‐pendrin (Figure 2C,D; Figure S3, Supporting Information). WT‐CFTR and WT‐pendrin each minimally interacted with TMED3 under normal conditions, with the interactions being increased by ARF1‐Q71L (Figure S4, Supporting Information). In both cases, ARF1‐Q71L blocked the Golgi maturation of CFTR and pendrin, and their ER forms were principally responsible for interaction with TMED3.

The relationship between TMED3 and ΔF508‐CFTR was also examined using immunofluorescence analyses with DYK‐tagged TMED3 (Figure 2E,F). HeLa cells were used instead of HEK293 cells for the morphological assay because they attach more firmly to coverslips and are less vulnerable to cell loss and disruption during the immunostaining procedures.^[^ ^6^ ^]^ In control cells, a TMED3 fraction of ≈40% colocalized with ΔF508‐CFTR. Under blocked ER‐to‐Golgi transport conditions, >70% of TMED3 proteins colocalized with ΔF508‐CFTR (Figure 2F), which further supports the notion that TMED3 participates in UPS. We then performed the cell surface biotinylation of TMED3 to examine whether TMED3 travels to the cell surface. As shown in Figure S5, Supporting Information, a negligible amount of TMED3 travelled to the cell surface under control and ARF1‐Q71L‐induced UPS conditions. Treatments with either the proteasome inhibitor MG132 or the dynamin inhibitor dynasore which inhibits vesicular endocytosis did not affect the cell surface expression of TMED3. Interestingly, the lysosomal V‐type H^+^‐ATPase inhibitor bafilomycin A1 significantly increased the cell surface expression of TMED3 under the ARF1‐Q71L‐induced UPS condition (Figure S5, Supporting Information). It thus appears that inhibition of lysosomal function augments and alters the UPS process, resulting in the plasma membrane transport of cargo‐associated molecules such as TMED3.

**Figure 3:**
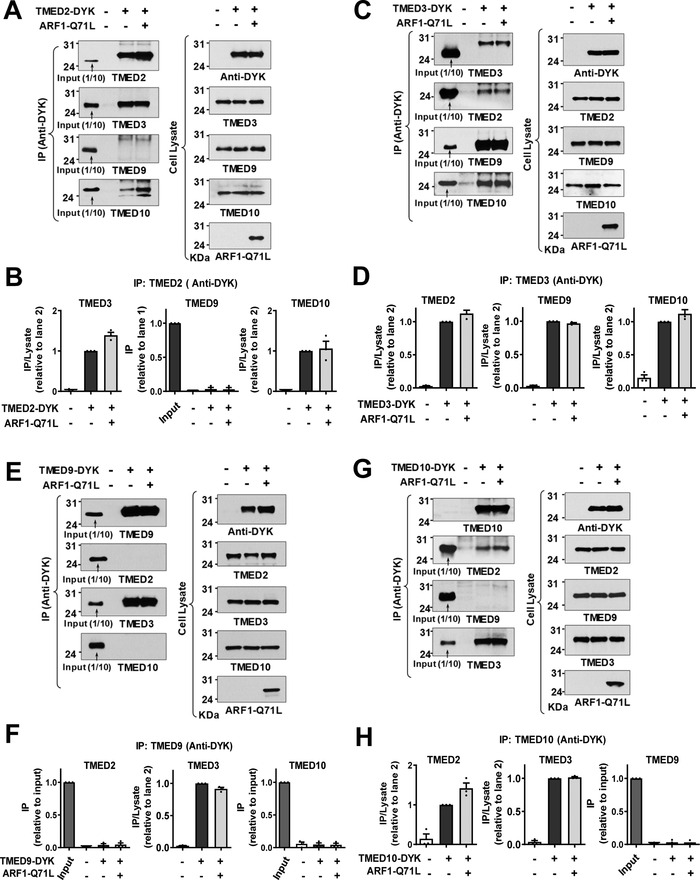
TMED3 is the key protein in the assembly of TMED2/3/9/10 complex. A,B) Co‐immunoprecipitation (co‐IP) experiments were performed with anti‐DYK antibodies in HEK293 cells transfected with plasmids encoding DYK‐tagged TMED2 (TMED2‐DYK). ARF1‐Q71L was co‐expressed in some cells to induce UPS. Protein samples were blotted with antibodies against each labeled protein. Summarized results of multiple experiments are presented in (B) (*n* = 3). TMED2 associates with TMED3 and TMED10. C,D) Co‐IP experiments were performed with plasmids encoding DYK‐tagged TMED3 (TMED3‐DYK). Summarized results of multiple experiments are presented in (D) (*n* = 3). TMED3 associates with TMED2, TMED9, and TMED10. E,F) Co‐IP experiments were performed with plasmids encoding DYK‐tagged TMED9 (TMED9‐DYK). Summarized results of multiple experiments are presented in (F) (*n* = 3). TMED9 associates with TMED3 but not with other TMEDs. G,H) Co‐IP experiments were performed with plasmids encoding DYK‐tagged TMED10 (TMED10‐DYK). Summarized results of multiple experiments are presented in (H) (*n* = 3). TMED10 associates with TMED2 and TMED3. Data are shown as mean ± SEM.

It has been suggested that TMED proteins in general function as stable heteromeric complexes.^[^ ^22^ ^]^ We then analyzed interactions among TMED proteins using the exogenous expression of DYK‐tagged TMED proteins. While TMED3 interacted with TMED2, TMED9 and TMED10, other TMEDs did not interact with each other directly except in the case of TMED2‐TMED10 interaction (**Figure** **3**). Taken together, these results indicate that TMED3 may act as the central regulator of CFTR and pendrin UPS by recognizing the ER‐accumulated cargos and bridging other TMED proteins required for ER stress‐associated protein secretion.

**Figure 4:**
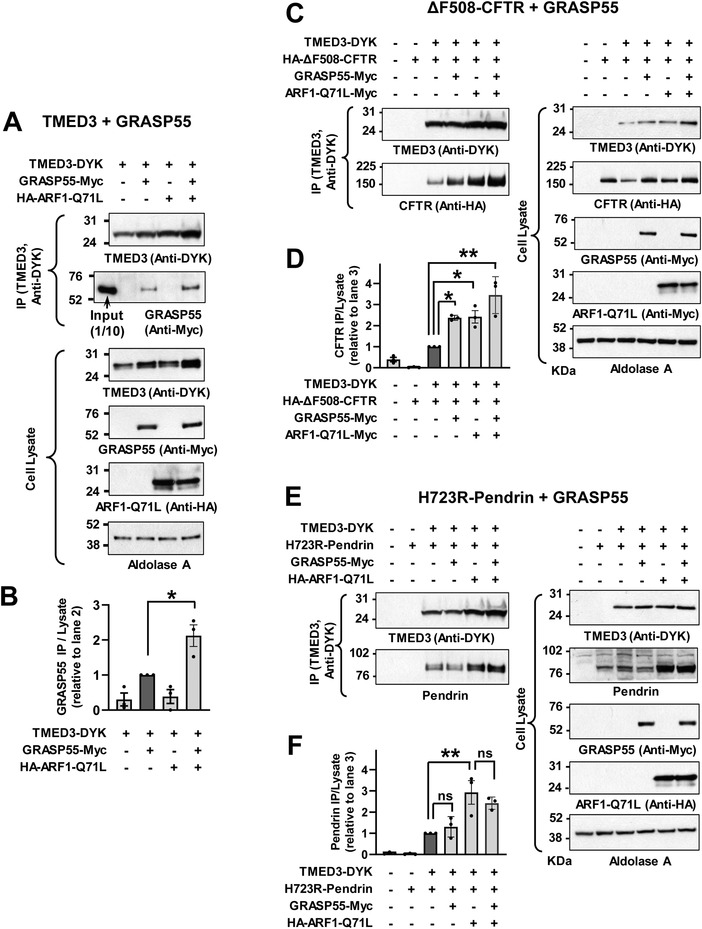
GRASP55 interacts with TMED3 and augments TMED3‐CFTR interaction but not TMED3‐pendrin interaction. A,B) Co‐immunoprecipitation (co‐IP) experiments with TMED3 and GRASP55 were performed with anti‐DYK antibodies in HEK293 cells transfected with plasmids encoding DYK‐tagged TMED3 (TMED3‐DYK) alone or those with GRASP55 (GRASP55‐Myc). ARF1‐Q71L was co‐expressed in some cells to induce UPS. Representative blot images are shown in (A) and summarized results of multiple experiments are presented in (B) (*n* = 3). ARF1‐Q71L enhanced interaction between TMED3 and GRASP55. C,D) Co‐IP experiments with TMED3 and ΔF508‐CFTR were performed with HA‐tagged ΔF508‐CFTR expression. GRASP55 and/or ARF1‐Q71L were co‐expressed in some cells. Representative blot images are shown in (C) and summarized results of multiple experiments are presented in (D) (*n* = 3). GRASP55 augmented the association between TMED3 and ΔF508‐CFTR. E,F) Co‐IP experiments with TMED3 and p.H723R‐pendrin were performed in PANC‐1 cells. GRASP55 and/or ARF1‐Q71L were co‐expressed in some cells. Representative blot images are shown in (E) and summarized results of multiple experiments are presented in (F) (*n* = 3). ARF1‐Q71L, but not GRASP55, increased the interaction between TMED3 and p.H723R‐pendrin. Bar graph data are shown as mean ± SEM. Data were analyzed using one‐way analysis of variance, followed by Tukey's multiple comparison test. **p < 0.01, ns: not significant.

The GRASP proteins are involved in the UPS of many cargo proteins including CFTR.^[^ ^4^ ^]^ As shown in **Figure** **4**A,B, TMED3 interacted with GRASP55 and this interaction was accelerated by ARF1‐Q71L. Interestingly, exogenous GRASP55 augmented the interaction between TMED3 and ΔF508‐CFTR (Figure 4C,D), but not that between TMED3 and p.H723R‐pendrin (Figure 4E,F), which is consistent with the previous results describing that knockdown of GRASP55 reduced the UPS of CFTR^[^ ^4^ ^]^ but not pendrin.^[^ ^5^ ^]^ For an underlying mechanism, the PDZ1 domain of GRASP55 has been shown to interact with the C‐terminal PDZ binding motif of CFTR^[^ ^4^, ^23^ ^]^ which is not present in the pendrin. Therefore, it appears that additional interaction with GRASP55 may facilitate the recruitment of CFTR in the TMED3‐mediated protein complex.

### 2.3 TMED3 Interacts with N‐Glycosylated CFTR and Pendrin through its GOLD Domain

**Figure 5:**
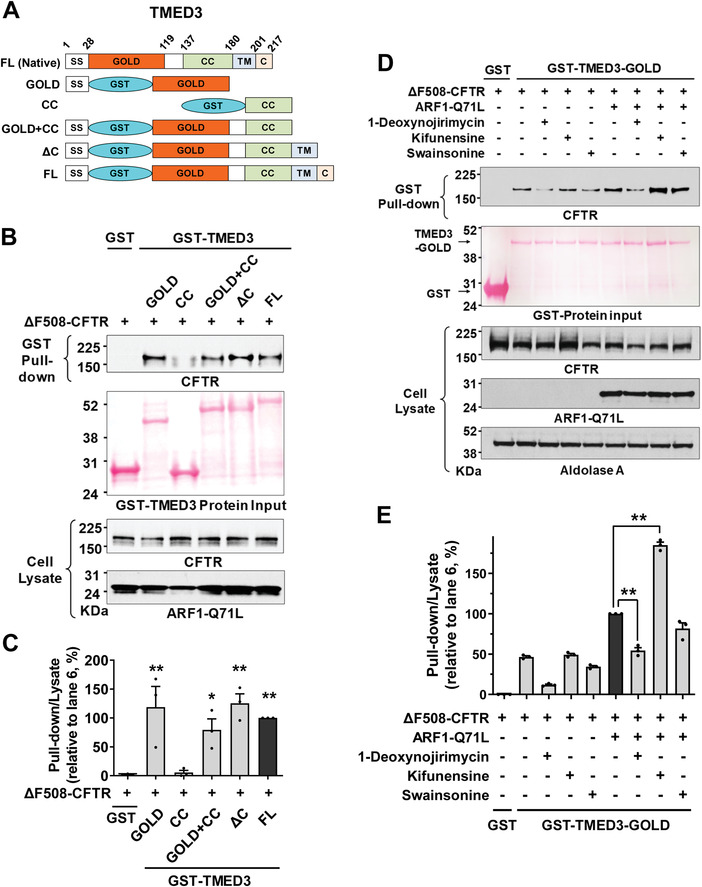
The GOLD domain of TMED3 binds to N‐glycosylated CFTR. A–C) Pull‐down assays were performed with domain‐specific GST‐fused TMED3 proteins (100 µg) and cell lysates extracted from HEK293 cells expressing HA‐tagged CFTR (400 µg). Schematic diagrams of GST‐tagged TMED3 proteins are depicted in (A). The input GST‐fused recombinant proteins were visualized using Ponceau S staining and CFTR was immunoblotted with anti‐HA antibodies. Representative pull‐down assays are shown in (B) and the results of multiple experiments are summarized in (C) (*n* = 3). The GOLD domain of TMED3 interacts with CFTR. **p < 0.01: difference from lane 1. D,E) Effects of glucosidase and mannosidase inhibitors on the interaction between TMED3 and ΔF508‐CFTR. Pull‐down assays were performed using recombinant GST‐fused TMED3‐GOLD (GST‐TMED3‐GOLD) and cell lysates prepared from HEK293 cells expressing ΔF508‐CFTR treated with the ER *α*‐glucosidase I and II inhibitor 1‐deoxynojirimycin (100 µm), ER mannosidase I inhibitor kifunensine (10 µm), or Golgi mannosidase II inhibitor swainsonine (100 µm). ARF1‐Q71L was co‐expressed in some cells to induce UPS. Representative pull‐down assays are shown in (D) and results of multiple experiments are summarized in (E) (*n* = 3). The inhibition of ER *α*‐glucosidase reduces the TMED3‐CFTR interaction; but the inhibition of ER mannosidase increases the TMED3‐CFTR interaction. Bar graph data are shown as mean ± SEM. **p < 0.01. Data were analyzed using one‐way analysis of variance, followed by Tukey's multiple comparison test.

We further explored the interactions between TMED3 and CFTR using a pull‐down assay to precisely identify the binding region of TMED3. Domain‐specific GST‐tagged TMED3 proteins were purified in vitro and incubated with protein samples from cells expressing HA‐tagged ΔF508‐CFTR. The domain structures of TMED3 proteins (FL, full length; SS, signal sequence; GST, glutathione‐S‐transferase; GOLD, Golgi dynamics domain; CC, coiled‐coil domain; TM, transmembrane domain; C, carboxy‐terminal tail) used in pull‐down assays are illustrated in **Figure** **5**A. Pull‐down assays with TMED3 recombinant proteins indicated that the GOLD domain of TMED3 (TMED3‐GOLD, a.a. 29–119) is principally responsible for its association with CFTR (Figure 5B,C). Additionally, pull‐down assays with p.H723R‐pendrin also revealed that the TMED3 GOLD domain binds to pendrin (Figure S6, Supporting Information).

We next examined the effects of ER glucosidase and mannosidase inhibitors on the association between CFTR and the TMED3 GOLD domain using pull‐down assays. As shown in Figure 5D,E, the inhibition of ER glucosidases by 1‐deoxynojirimycin significantly reduced TMED3‐CFTR interaction, suggesting that TMED3 principally binds to CFTR proteins that have a deglucosylated form of N‐glycans. In contrast, treatment with the ER mannosidase inhibitor kifunensine increased TMED3‐CFTR interaction, implying that TMED3 preferentially binds to CFTR with high‐mannose glycans. Treatment with the Golgi mannosidase inhibitor swainsonine did not significantly affect the association between CFTR and TMED3 (Figure 5D,E). Collectively, these results imply that TMED3 mostly targets cargo proteins that contain high‐mannose type N‐glycans (see Discussion).

### 2.4 Combined Expression of TMED2/3/9/10, but not Single TMED Expression, Enhances the UPS of ΔF508‐CFTR and p.H723R‐Pendrin

**Figure 6:**
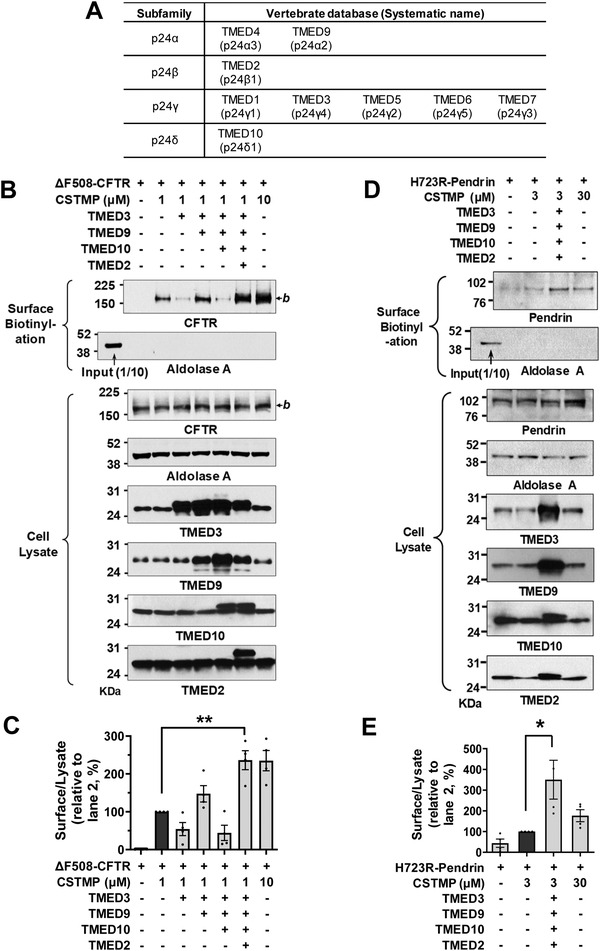
Potentiation of CFTR and pendrin UPS by TMED upregulation. A) Classification of vertebrate TMED subfamilies, also known as p24 proteins. B,C) The effects of exogenous expression of TMED proteins (TMED2, 3, 9, and 10) on the CSTMP‐induced UPS of ΔF508‐CFTR were examined using the surface biotinylation assay. Representative blots are shown in (B) and results of multiple experiments are summarized in (C) (*n* = 4). Co‐expression of all four TMEDs significantly increased a low‐dose (1 *μ* m) CSTMP‐induced ΔF508‐CFTR UPS. D,E) The effects of exogenous expression of TMED proteins (TMED2, 3, 9, and 10) on the CSTMP‐induced UPS of p.H723R‐pendrin were examined using the surface biotinylation assay. Representative blots are shown in (D) and results of multiple experiments are summarized in (E) (*n* = 4). Co‐expression of all four TMEDs significantly increased a low‐dose (3 *μ* m) CSTMP‐induced p.H723R‐pendrin UPS. Bar graph data are shown as mean ± SEM. *p < 0.05, **p < 0.01. Data were analyzed using one‐way analysis of variance, followed by Tukey's multiple comparison test.

The ten‐membered TMED proteins can be classified into four subfamilies and are known to form a heterotetrameric complex containing each member of the four subfamilies (*α*, *β*, *γ*, and *δ*; **Figure** **6**A).^[^ ^22^ ^]^ Interestingly, overexpression of TMED3 (p24*γ*4) alone inhibited, while co‐expression with TMED9 (p24*α*2) restored, the CSTMP‐induced cell surface expression of ΔF508‐CFTR (Figure 6B,C; lanes 3 and 4) suggesting that the *αγ* heteromeric TMEDs play a role in CFTR UPS. Similarly, exogenous TMED10 (p24*δ*1) addition inhibited the ΔF508‐CFTR UPS, while co‐addition of TMED2 (p24*β*1) restored it (Figure 6B,C; lanes 5 and 6), implying that the *βδ* TMED heteromers participate in CFTR UPS. Most importantly, co‐expression of all four TMEDs substantially increased a low‐dose CSTMP‐induced (1 *μ* m) ΔF508‐CFTR UPS, which reached the level ordinarily induced by a tenfold higher CSTMP concentration (10 *μ* m) (Figure 6B,C; lanes 6 and 7).

We then examined the effects of exogenous TMEDs on the UPS of p.H723R‐pendrin. As has also been shown in the case of ΔF508‐CFTR, exogenous single expressions of TMED2, TMED3, TMED9, and TMED10 proteins reduced the UPS of p.H723R‐pendrin (Figure S7, Supporting Information). In contrast, co‐expression of all four TMEDs (TMED2, 3, 9, and 10) strongly augmented the low dose CSTMP (3 µm)‐induced cell surface expression of p.H723R‐pendrin, which reached the level induced by a tenfold higher CSTMP concentration (30 *μ* m) (Figure 6D,E). The above results suggest that TMEDs function as a heteromultimeric complex and that the stoichiometric balances are critical to the proper functioning of transmembrane protein UPS.

### 2.5 Co‐Expression of TMED2/3/9/10 Restores Ion Transport Function in ΔF508‐CFTR and p.H723R‐Pendrin

**Figure 7:**
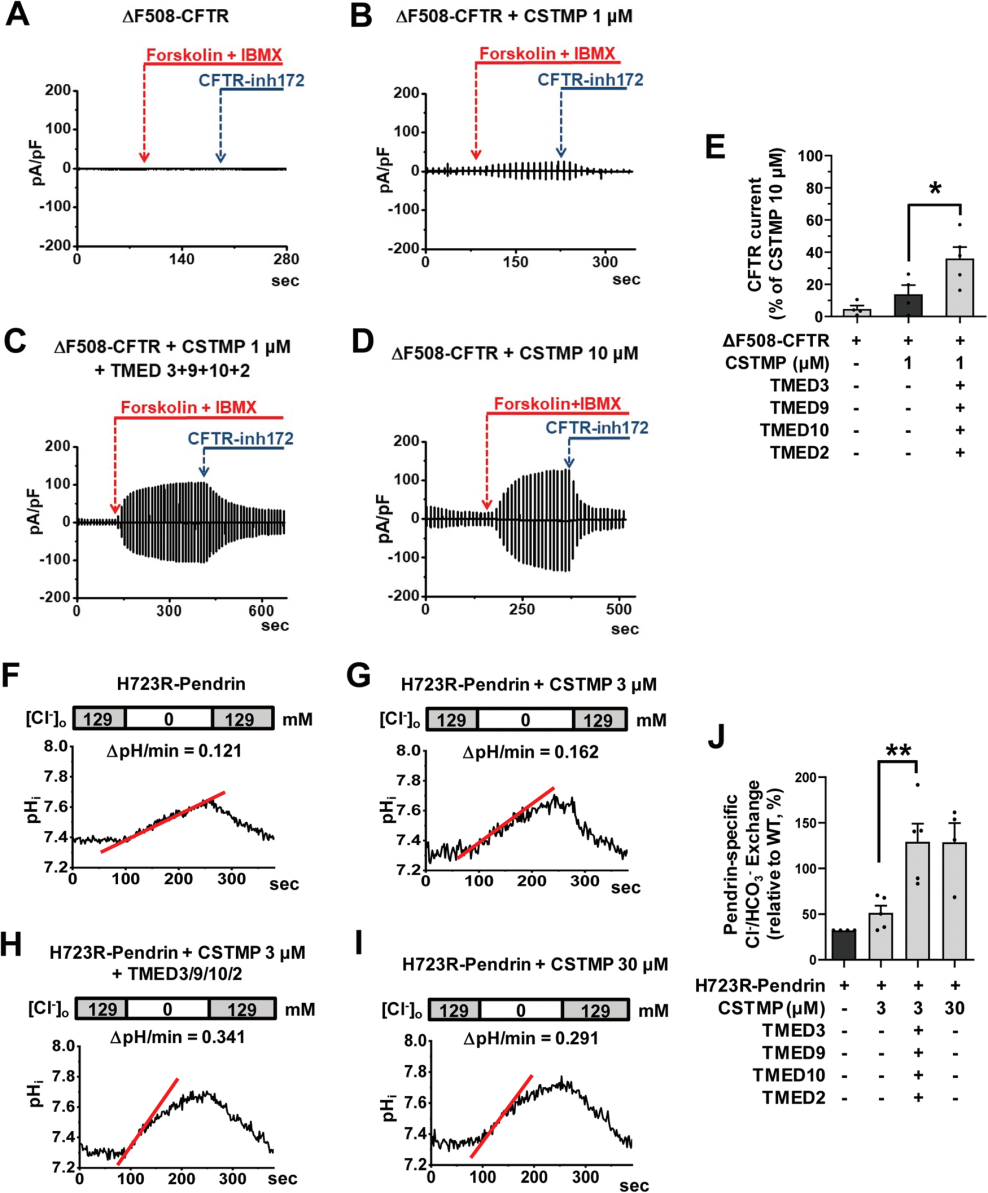
Functional rescue of ΔF508‐CFTR and p.H723R‐pendrin by TMED upregulation. A–E) Cl^−^ currents were measured with co‐expression of TMEDs in cells expressing ΔF508‐CFTR. The CFTR‐mediated currents were activated by cAMP treatment (forskolin and 3‐isobutyl‐1‐methylxanthine [IBMX]) and inhibited by CFTRinh‐172 (10 µm). Representative current recordings and *I*–*V* curves are shown in (A–D) and Figure S8A–D, Supporting Information, respectively. The results of multiple experiments are summarized in (E) (*n* = 4–5). Co‐expression of TMED2, 3, 9, and 10 significantly increased CFTR Cl^−^ currents in cells treated with a low dose (1 µm) CSTMP. F–J) The pendrin‐specific Cl^−^_o_/HCO_3_^−^_i_ exchange activity was measured by recording pH_i,_ as detailed in Figure S8E–G, Supporting Information. Representative anion exchange measurements are shown in (F–I) and the quantifications of multiple experiments are depicted in (J) (*n* = 4–5). Co‐expression of TMED2, 3, 9, and 10 significantly increased the pendrin‐specific anion exchange activity in cells treated with a low dose (3 µm) CSTMP. Bar graph data are shown as mean ± SEM. *p < 0.05, **p < 0.01. Data were analyzed using one‐way analysis of variance, followed by Tukey's multiple comparison test.

To identify the therapeutic potential of TMED‐mediated UPS, we examined how the exogenous expression of TMED proteins affects the ion transport function of the trafficking‐defective ΔF508‐CFTR and p.H723R‐pendrin mutants. We first evaluated the functional significance of TMEDs by measuring CFTR anion currents (**Figure** **7**A–E). The CFTR‐induced Cl^−^ currents were characterized by the following three findings: 1) activation by cAMP treatment (forskolin and 3‐isobutyl‐1‐methylxanthine [IBMX]); 2) inhibition by the CFTR inhibitor CFTRinh‐172; and 3) a linear *I*–*V* relationship (Figure S8A–D, Supporting Information). The cAMP treatment did not evoke discernible Cl^−^ currents in HEK293 cells expressing ΔF508‐CFTR, and the treatment with 1 µm CSTMP for 48 h induced a minimal level of CFTR Cl^−^ currents (Figure 7A,B). Importantly, co‐expression of TMED2, 3, 9, and 10 significantly increased CFTR Cl^−^ currents in cells treated with a low dose (1 µm) CSTMP (Figure 7C–E).

We then examined the effects of exogenous TMEDs on the anion exchange activity of pendrin. Notably, co‐expression of TMED2, 3, 9, and 10 strongly augmented the low dose CSTMP (3 µm)‐induced pendrin‐mediated Cl^−^/HCO_3_^−^ exchange activity in cells expressing p.H723R‐pendrin, reaching the level induced by a tenfold higher CSTMP concentration (30 *μ* m) (Figure 7F–J; Figure S8E–G, Supporting Information). These results indicate that TMED upregulation augments the UPS‐mediated functional restoration of ΔF508‐CFTR and p.H723R‐pendrin.

### 2.6 TMEDs Mediate ER Stress‐Associated Secretion of SARS‐CoV‐2 Spike

**Figure 8:**
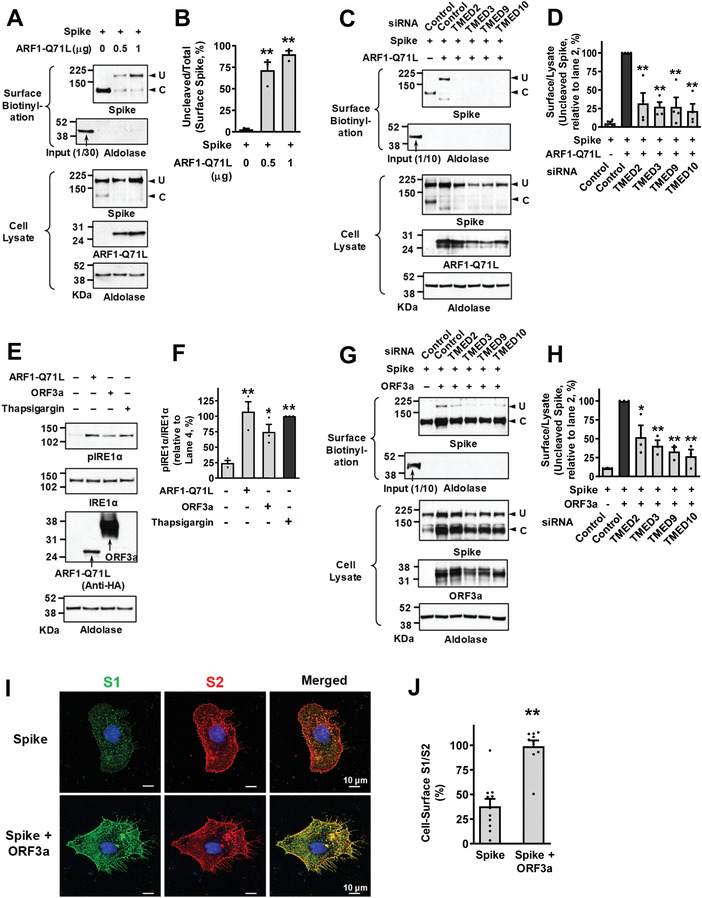
TMEDs mediate ER stress‐associated secretion of SARS‐CoV‐2 Spike. A,B) The ER‐to‐Golgi blockade induces UPS of SARS‐CoV‐2 Spike (S). The cell surface expression of uncleaved (U) and cleaved (C) S proteins was analyzed using the surface biotinylation assays in HEK293 cells. Plasmids encoding ARF1‐Q71L (0.5 or 1 *μ*g) were transfected to induce ER‐to‐Golgi blockade. Representative surface biotinylation assays are shown in (A) and the results of multiple experiments are summarized in (B) (*n* = 3). C,D) Silencing of TMED2, TMED3, TMED9, and TMED10 inhibits ARF1‐Q71L‐induced UPS of S. The effects of *TMED* gene silencing (100 nm each, 48 h) on the cell surface expression of S were analyzed. Representative blots of surface biotinylation assays are shown in (C) and the results of multiple experiments are summarized in (D) (*n* = 4). E,F) The SARS‐CoV‐2 ORF3a induces ER stress. Phosphorylation of IRE1*α*, a marker of ER stress, was analyzed in HEK293 cells transfected with ARF1‐Q71L and ORF3a (24 h). Thapsigargin (2 µm, 6 h) was used as a positive control to induce ER stress. Representative immunoblots are shown in (E) and the results of multiple experiments are summarized in (F) (*n* = 3). G,H) Silencing of TMED2, TMED3, TMED9, and TMED10 inhibits ORF3a‐induced UPS of S. The effects of *TMED* gene silencing (100 nm each, 48 h) on the cell surface expression of S were analyzed with co‐expression of SARS‐CoV‐2 ORF3a. Representative blots of surface biotinylation assays are shown in (G) and the results of multiple experiments are summarized in (H) (*n* = 3). I,J) The cell surface expression of the S1 (green) and S2 (red) fragments of S proteins was analyzed using immunocytochemistry in HeLa cells with co‐expression of ORF3a. Representative immunocytochemistry images are shown in (I) and the results of multiple experiments are summarized in (J) (*n* = 10–11). Bar graph data are shown as mean ± SEM. *p < 0.05, **p < 0.01. Data were analyzed using one‐way analysis of variance, followed by Tukey's multiple comparison test (B,D,F,H) or a two‐tailed Student's *t*‐test (J).

Because SARS‐CoV‐2 S is an N‐glycosylated transmembrane protein^[^ ^13^ ^]^ and because, like many viral infections, SARS‐CoV‐2 infection can trigger ER stress,^[^ ^16^ ^]^ we examined whether TMEDs affect the intracellular trafficking of SARS‐CoV‐2 S. Initially, we determined the cell surface trafficking of S in HEK293 cells transfected with plasmid vectors encoding SARS‐CoV‐2 S (**Figure** **8**). The SARS‐CoV‐2 S protein can be cleaved into S1 and S2 fragments by furin, a cellular protease enriched in the Golgi apparatus.^[^ ^24^ ^]^ A control experiment revealed that the Golgi localization of furin is not significantly affected during SARS‐CoV‐2 infection (Figure S9, Supporting Information). Exogenous expression of SARS‐CoV‐2 S produced two protein bands, representing the uncleaved full‐length S and cleaved S proteins, respectively (Figure 8A). Interestingly, almost all the cell surface S proteins under control conditions were in the cleaved S, indicating that they had mostly travelled through the conventional Golgi‐mediated route (Figure 8A,B). However, when the conventional route was blocked by ARF1‐Q71L, a significant amount of S could still reach the cell surface and >90% cell surface S were the uncleaved full‐length S (Figure 8A,B; 1 *μ*g ARF1‐Q71L transfection), suggesting that these proteins had reached the cell surface via a Golgi‐bypassing route. Importantly, this ARF1‐Q71L‐induced, Golgi‐independent trafficking of S was strongly inhibited by silencing of *TMED2*, *TMED3*, *TMED9*, and *TMED10* (Figure 8C,D), showing that TMEDs are also involved in the UPS of S. Among the TMED*γ* subfamily (TMED1, 3, 5, 6, and 7), TMED3 knockdown demonstrated the strongest effect in reducing the UPS of S (Figure S10A,B, Supporting Information). The immunoprecipitation results indicate that TMED3 is again principally responsible for associating with the cargo protein S (Figure S11, Supporting Information).

Among SARS‐CoV and SARS‐CoV‐2 proteins, ORF3a was shown to cause ER stress and ER reorganization.^[^ ^16^, ^25^ ^]^ The ORF3a is an accessory protein believed to function as a cation‐permeable viroporin and is associated with the induction of autophagy and apoptosis in host cells.^[^ ^26^ ^]^ As shown in Figure 8E,F, the expression of SARS‐CoV‐2 ORF3a evoked the phosphorylation of IRE1*α*, which is an indicator of ER stress and is known to play a key role in the ER stress‐induced UPS of CFTR and pendrin.^[^ ^6^ ^]^ Notably, co‐expression of ORF3a evoked a cell surface expression of uncleaved S (Figure 8G,H), indicating that ORF3a‐induced ER stress is associated with Golgi‐independent trafficking of S. While ARF1‐Q71L‐induced ER‐to‐Golgi blockade abolished the cell surface expression of cleaved S (Figure 8C), ORF3a did not (Figure 8G). In immunofluorescence analyses using antibodies against the S1 and S2 subunits, co‐expression of ORF3a increased the relative cell surface expression of S1 (S1/S2 ratio; Figure 8I,J). This may support the surface biotinylation finding that ORF3a increases the cell surface expression of full‐length S, since parts of cleaved S1 can be removed from the membrane‐anchored S2 even though S1 can still bind to S2 via a non‐covalent interaction.^[^ ^27^ ^]^ Importantly, silencing of *TMED2*, *3*, *9*, and *10* significantly inhibited the ORF3a‐induced transport of uncleaved S in the surface biotinylation assays (Figure 8G,H), revealing that TMEDs participate in the ORF3a‐induced, ER stress‐associated secretion of S. Interestingly, knockdown of GRASP55 also partially reduced the ORF3a‐induced UPS of S (Figure S10C,D, Supporting Information).

**Figure 9:**
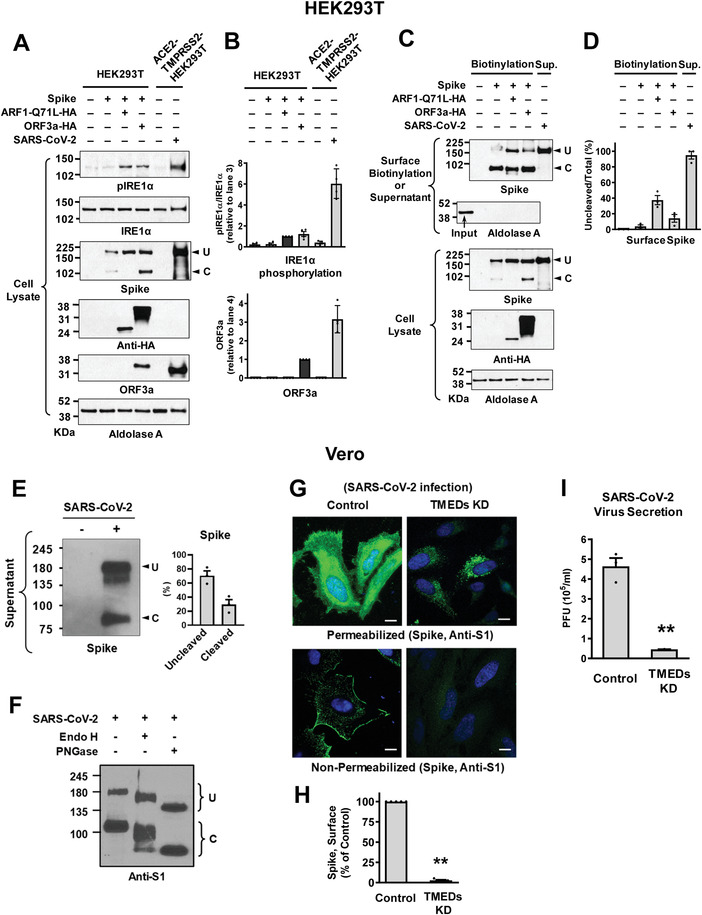
Trafficking of S in the authentic SARS‐CoV‐2 viruses. A,B) IRE1*α* phosphorylation and ORF3a expression were analyzed in HEK293T cells transfected with the indicated plasmids (lanes 1–4) or those with authentic SARS‐CoV‐2 infection (lane 6). HEK293T cells stably expressing ACE2 and TMPRSS2 (ACE2‐TMPRSS2‐HEK293T) were used for the SARS‐CoV‐2 infection. Representative immunoblots detected with anti‐phospho IRE1*α* or anti‐ORF3a are shown in (A) and the results of multiple experiments are summarized in (B) (*n* = 3–4). The authentic SARS‐CoV‐2 virus infection evoked higher levels of ORF3a expression and IRE1*α* phosphorylation than the ORF3a plasmid transfection. C,D) The expression of SARS‐CoV‐2 S in the cell surface or viral particle was analyzed. The cell surface biotinylation was performed in HEK293T cells transfected with the indicated plasmids (Biotinylation). The SARS‐CoV‐2 virions were harvested from the supernatant of infected ACE2‐TMPRSS2‐HEK293T cells (Sup). Representative immunoblots are shown in (C) and the results of multiple experiments are summarized in (D) (*n* = 4). The SARS‐CoV‐2 virions contain a higher level of uncleaved S. E) The amounts of uncleaved (U) and cleaved (C) S in the SARS‐CoV‐2 virus particles were analyzed. Proteins samples harvested from the supernatant of infected Vero cell cultures (SARS‐CoV‐2 KUMC‐2, GISAID accession#: EPI_ISL_413 018; 0.01 MOI, 48 h) were blotted with antibodies against the S2 subunit. Representative immunoblots and the results of multiple experiments are summarized in (E) (*n* = 3). The authentic SARS‐CoV‐2 virions contained >50% uncleaved S. F) The glycosylation status of S was analyzed via digestion with endoglycosidase H (Endo H) and N‐glycosidase F (PNGase F). SARS‐CoV‐2 contains partially Endo H‐sensitive, hybrid types of N‐glycans. Results shown are representative of three independent experiments. G,H) Cell localization of S was analyzed using immunocytochemistry in permeabilized and non‐permeabilized Vero cells infected with SARS‐CoV‐2 (10 h post‐infection). Quantification of cell surface intensity of S is summarized in (H) (*n* = 5). Scale bar: 10 µm. I) Viral RNA in cell culture supernatant was analyzed using quantitative PCR. Vero cells were infected with 0.01 MOI SARS‐CoV‐2, then culture supernatant was harvested at 24 h post‐infection. Bar graph data are shown as mean ± SEM. **p < 0.01. Data were analyzed using a two‐tailed Student's *t*‐test (H,I).

We next examined the trafficking of S in the authentic SARS‐CoV‐2 viruses. In the first set of experiments, HEK293T cells were used for transfection of plasmids and HEK293T cells stably expressing ACE2 (the receptor for S) and TMPRSS2 (the S activating enzyme) were used for SARS‐CoV‐2 infections. Notably, SARS‐CoV‐2 infections evoked a 3.2‐fold higher level of ORF3a expression than the ORF3a plasmid transfection, which resulted in a much stronger phosphorylation of IRE1*α* (**Figure** **9**A,B). This strong ER stress activation was associated with a higher level of uncleaved S in the SARS‐CoV‐2 virion harvested from the supernatant of infected cells (Figure 9C,D).

The SARS‐CoV‐2 virions were also harvested from the supernatant of infected Vero cells that endogenously express ACE2. In contrast to the results for the exogenous expression of S using plasmids, in which <5% of cell surface S were uncleaved under control conditions (Figure 8A,B), >70% of S in the secreted SARS‐CoV‐2 virions were uncleaved (Figure 9E). This indicates that a large fraction of SARS‐CoV‐2 viruses are secreted via a Golgi‐bypassing route. We then characterized the N‐glycosylation status of S via digestion with endoglycosidase H (Endo H) and N‐glycosidase F (PNGase F). Endo H removes the ER‐mediated high mannose and some hybrid types of N‐linked carbohydrates but not the Golgi‐mediated complex oligosaccharides.^[^ ^28^ ^]^ The results in Figure 9F show that S in the SARS‐CoV‐2 virion contains partially Endo H‐sensitive, hybrid types of N‐glycans, further indicating that the secreted SARS‐CoV‐2 does not pass through the entire compartments of the Golgi stacks. Notably, silencing of *TMED2/3/9/10* evoked intracellular retention of S in the Vero cells infected with SARS‐CoV‐2 (Figure 9G,H). Lastly, silencing of TMEDs induced a 90% reduction in the secreted viral titers in Vero cells infected with SARS‐CoV‐2 (Figure 9I), implying that inhibition of TMEDs could be a potential therapeutic target for the development of antiviral agents against SARS‐CoV‐2.

## 3 Discussion

The accumulation of unfolded proteins in the ER causes cellular stress, triggering an unfolded protein response to deescalate the protein burden by reducing transcription and translation of secretory proteins and by directing misfolded proteins into ERAD.^[^ ^29^ ^]^ In addition to these classical mechanisms, cells trigger UPS to diminish the accumulated protein burden in the ER^[^ ^4^ ^]^ and cytoplasm.^[^ ^30^ ^]^ Over the last two decades, researchers have incrementally established the concept and molecular mechanisms for the non‐canonical UPS of various proteins, including transmembrane proteins.^[^ ^3^, ^31^ ^]^ For example, it has been shown that GRASPs, heat shock proteins and their cochaperones, and autophagy components are involved in the UPS of various transmembrane proteins, such as CFTR, pendrin, Mpl, and *α*‐integrins.^[^ ^4^, ^5^, ^32^, ^33^, ^34^ ^]^ However, key questions concerning how these ER membrane cargos are sorted for UPS remain unresolved. In the present study, we show that the TMED cargo receptor family members TMED2, 3, 9, and 10 participate in the ER stress‐associated UPS of transmembrane proteins and that, in particular, TMED3 (also known as p26 or p24*γ*4) plays a key role in recognizing the ER transmembrane cargo proteins.

TMED family proteins have been implicated in protein transport between the ER‐Golgi interfaces,^[^ ^35^ ^]^ initially being considered a cargo receptor for the incorporation of secretory cargo into COPII or COPI vesicles.^[^ ^36^ ^]^ Because the transport of some cargo proteins (i.e., GPI‐anchored proteins, the tsO45 mutant of VSV‐G and Gas1p) were inhibited by individually depleted TMED proteins but others were not (i.e., acid phosphatase and alkaline phosphatase),^[^ ^35^, ^37^, ^38^ ^]^ each TMED member probably has a unique specificity as a cargo receptor. Notably, knockdown of TMEDs (TMED2, 3, 9, and 10) significantly reduced the stress‐induced UPS of CFTR, pendrin, and SARS‐CoV‐2 S (Figures 1 and 8) but did not noticeably affect their conventional cell surface transport (Figure 8G; Figure S2, Supporting Information). Therefore, although TMEDs may function also in the conventional transport of some other cargos, the TMED‐mediated pathway appears to be a dominant route for the ER exit of CFTR, pendrin, SARS‐CoV‐S under cellular stress conditions. In the current study, we employed a dominant‐inhibitory form of ARF1 (ARF1‐Q71L) to block ER‐to‐Golgi transport and induce UPS. It has been shown that TMED proteins are involved in the biogenesis of COPI vesicles and interact with ARF1‐GDP,^[^ ^35^ ^]^ raising the possibility that the present effect of TMED might be confined to the ARF1 mutant‐induced UPS. However, this is unlikely because the TMED proteins also participate in CFTR UPS induced by the IRE1*α* kinase activator CSTMP (Figure 1C,D) and in the ORF3a‐induced trafficking of SARS‐CoV‐2 S (Figure 8G,H). Furthermore, the reported TMED‐COPI interaction appears irrelevant to the present finding, since it plays a functional role in retrograde Golgi‐to‐ER trafficking^[^ ^35^ ^]^ but not in the trafficking from the ER.

In general, TMED proteins function as a multimeric complex. A number of reports suggest that heterotetrameric complexes composed of *αγ* and *βδ* dimers play a major role in TMED‐mediated vesicular transport.^[^ ^22^ ^]^ The results in the present study also support this notion. While single overexpression of TMED3 (p24*γ*4) and TMED10 (p24*δ*1) resulted in a significant reduction in CFTR UPS, the supplementation of TMED9 (p24*α*2) and TMED2 (p24*β*1), respectively, led to a recovery in it (Figure 6B,C). This suggests that TMEDs function as *αγ* and *βδ* dimers and that the stoichiometric imbalances of *α*:*γ* and *β*:*δ* subfamilies lead to a deterioration in TMED function. Interestingly, the double overexpression of *αγ* (TMED9 and TMED3) subfamilies did not inhibit CFTR UPS (Figure 6B,C; lane 4), implying that stoichiometric balance between *αγ* and *βδ* dimers is not strictly required. It appears that the TMED*γ* subfamily (TMED1, 3, 5, 6, and 7) may contribute to the substrate specificity. Although TMED3 knockdown showed the strongest effect, knockdown of TMED1 also reduced the UPS of pendrin (Figure 1E,F) and knockdowns of TMED1 and TMED5 partially reduced the UPS of S (Figure S10A,B, Supporting Information). Therefore, diverse TMED*γ* proteins may recruit their own specific substrates to the TMED complex for intracellular protein trafficking. A recent study reported that TMED10 mediates the UPS of a soluble cytosolic cargo IL‐1*β*, perhaps by forming a homo‐multimeric channel.^[^ ^39^ ^]^ However, the study examined neither the relationship between TMED10 and other TMEDs nor the role of other TMED proteins in IL‐1*β* UPS. Further investigations are required to identify the mechanistic details underlying TMED10‐mediated IL‐1*β* UPS.

The immunoprecipitation results revealed that TMED3 is primarily responsible for recruiting the N‐glycosylated membrane proteins, including CFTR, pendrin and SARS‐CoV‐2 S, into the TMED complex (Figure 2A–D; Figure S11, Supporting Information). The pull‐down experiments with inhibitors of ER glucosidases and mannosidases showed that TMED3 preferentially binds to cargos that contain deglucosylated high‐mannose glycans (Figure 5D,E). When newly synthesized polypeptides enter the ER lumen, oligosaccharides composed of three glucoses (Glc_3_), nine mannoses (Man_9_), and two N‐acetylglucosamines (GlcNAc_2_) are attached to the asparagine residues of polypeptides. The Glc_3_Man_9_GlcNAc_2_ glycans are then trimmed to the deglucosylated Man_9_GlcNAc_2_ glycans by ER glucosidases and further trimmed to Man_8_GlcNAc_2_ glycans by ER mannosidases.^[^ ^40^ ^]^ Collectively, these findings imply that Man_9_GlcNAc_2_ appears to be the prime substrate of TMED3.

Transmembrane proteins that fail to pass ERQC are selectively targeted for ERAD to prevent damage caused by malfunctioning proteins when they escape the ER.^[^ ^29^ ^]^ On the other hand, mutations that cause folding defects in membrane proteins often result in autosomal‐recessive, loss‐of‐function diseases due to the absence of functional proteins at the cell surface. Many of the disease‐causing mutations in the membrane protein, including ΔF508‐CFTR and p.H723R‐pendrin, cause human diseases via this pathogenic mechanism. In the typical glycan trimming pathway, the removal and addition of sugar moieties on N‐glycans are an important determinant of ERQC and ERAD. For example, N‐glycan conversion to shorter mannose‐containing chains (e.g., Man_7_GlcNAc_2_) by ER degradation‐enhancing *α*‐mannosidase‐like proteins (EDEMs) directs misfolded N‐glycoproteins to ERAD. In addition, re‐monoglucosylation of N‐glycoproteins by UDP‐glucose glycoprotein glucosyltransferase (UGGT) promotes protein folding via re‐entry into the calnexin/calreticulin cycle.^[^ ^41^ ^]^ Because TMED3 principally binds to cargos that contain deglucosylated high‐mannose glycans (e.g., Man_9_GlcNAc_2_‐CFTR), it can efficiently hijack the ER‐accumulated membrane cargos for UPS, before reglucosylation‐induced re‐entry into the calnexin cycle or mannose trimming‐induced degradation under ER stress conditions. The SARS‐CoV‐2 S associates with TMED3 in control cells as well as in ARF1‐Q71L expressing cells (Figure S11B, Supporting Information), which may be related to the glycosylation pattern of S. While CFTR and pendrin each contain only two N‐linked glycosylation sites, the SARS‐CoV‐2 S contains 22 such sites with multiple oligomannose‐type glycans^[^ ^13^ ^]^ which may facilitate interaction with TMED3. However, TMED3 appears to be dispensable or redundant to the Golgi‐mediated trafficking, since TMED3 silencing did not noticeably affect the cell surface trafficking of furin‐cleaved S while it strongly inhibited the surface trafficking of uncleaved S (Figure 8G,H).

The present results indicate that TMEDs are required for the IRE1*α*‐mediated UPS of CFTR (Figure 1C,D), raising the question of how IRE1*α* regulates TMEDs. It would be interesting to examine whether post‐translational mechanisms, such as TMED phosphorylations, are involved in the TMED‐UPS cargo association. At present, it is not known whether TMEDs are phosphorylated by IRE1*α* or other kinases. It has been shown that deletion of *erv25* (yeast *TMED10* homolog) activates the unfolded protein response in yeast^[^ ^42^ ^]^ and that knockdown of TMED2 enhances the phosphorylation of IRE1*α* in mammalian THP‐1 cells,^[^ ^43^ ^]^ suggesting that multiple levels of cross regulation between TMEDs and IRE1*α* signaling exist. Further studies that analyze the IRE1*α*‐induced post‐translational modifications of TMEDs and the full repertoires of associating proteins in the TMED complex may contribute to the resolution of this question. Interestingly, TMED3 interacted with GRASP55 and augmented the recruitment of GRASP‐dependent cargos, such as CFTR,^[^ ^4^, ^23^ ^]^ into the TMED complex (Figure 4). This may in part explain the role of GRASP proteins in the UPS process. It is also of interest that the ORF3a‐induced UPS of SARS‐CoV‐2 S was significantly reduced by GRASP55 knockdown (Figure S10C,D, Supporting Information). The underlying mechanism of how GRASP55 is involved in the UPS of S awaits further investigation. In general, GRASP proteins recruit their membrane cargos via the PDZ‐based interactions.^[^ ^4^, ^23^ ^]^ Although SARS‐CoV‐2 S does not have the classical PDZ binding motif, it has been shown that a C‐terminal tail region of S interacts with the PDZ domain of SNX27.^[^ ^44^, ^45^ ^]^

A notable finding in this study is that TMED upregulation potentiated the CSTMP‐induced UPS of ΔF508‐CFTR and p.H723R‐pendrin. When combined with TMED upregulation, a 1/10 dose (1–3 *μ* m) of CSTMP showed similar effects to a full dose (10–30 *μ* m) in inducing the UPS of CFTR and pendrin (Figures 6 and 7). A sustained and strong activation of IRE1*α* could aggravate epithelial inflammation, although treatments with CSTMP at 10 µm concentration did not cause acute toxicity in HEK293 cells or in mice.^[^ ^6^ ^]^ Therefore, a reduced CSTMP dose along with co‐activation of TMED function would alleviate potential CSTMP toxicity and increase clinical applicability to the treatment of human diseases.

The finding that knockdown of TMEDs reduced intact virion secretion to the extracellular space (Figure 9I) indicates that TMED inhibition could provide a potential pharmacological target against SARS‐CoV‐2 infections. Interestingly, SARS‐CoV‐2 S does not appear to travel solely through the Golgi‐independent UPS route. Studies examining the glycosylation patterns of extracellular SARS‐CoV‐1 and SARS‐CoV‐2 S proteins have demonstrated that their glycosylation patterns are heterogenous and include hybrid/complex glycans.^[^ ^13^, ^46^ ^]^ In addition, ≈30% of S proteins in the SARS‐CoV‐2 virions were furin‐cleaved (Figure 9E). These results indicate that either they travel through multiple routes or they at least in part contact Golgi‐originated vesicles during their journey to the cell surface. The S1 subunit contains the ACE2‐binding domain and the S2 subunit incorporates the transmembrane domain that anchors in the viral envelope. Although S1 can still associate with S2 after cleavage via non‐covalent interactions,^[^ ^27^ ^]^ a complete pre‐cleavage of S may weaken the viral infectivity by reducing the ACE2‐binding ability. A recent study reported that *β*‐coronaviruses use an endo‐lysosomal system for their egress instead of the brefeldin A‐sensitive, conventional secretory pathway.^[^ ^47^ ^]^ However, the early secretory pathway via which the S proteins in the ER target to the viral assembly site and lysosomal exocytosis is yet to be identified. During viral infections, secretory tubular and vesicular systems, including the ER and the Golgi apparatus, undergo disassembly and reorganization,^[^ ^16^, ^17^ ^]^ which may result in the redistribution of their contents and provide additional secretory routes. Although further study is warranted to fully identify the secretory route(s) of SARS‐CoV‐2 S, the present results indicate that TMEDs play an important role in its secretion, and that, in particular, TMED3 is a key molecule that recognizes SARS‐CoV‐2 S to facilitate its ER exit.

In conclusion, our investigation provides evidence for the common role of TMEDs in the ER stress‐associated secretion of CFTR, pendrin, and SARS‐CoV‐2 S and for the identification of TMED3 as a receptor for transmembrane protein cargos. Collectively, these findings shed light on the general mechanism for how transmembrane proteins in the ER can be targeted to the secretion pathway away from degradation by ERAD under conditions of cellular stress. Given that modulating the intracellular trafficking of transmembrane proteins has therapeutic potential for various human diseases, including CF, Pendred syndrome and COVID‐19, these findings may have considerable implications in the identification of new strategies to treat diseases that are associated with the defective or harmful secretion of membrane proteins.

## 4 Experimental Section

### Cell Culture

HEK293, HEK293T, PANC‐1, and HeLa cells were maintained in Dulbecco's modified Eagle's medium‐high glucose (Gibco #11995‐065, Carlsbad, CA) supplemented with 10% fetal bovine serum (FBS) and 1% 100× antibiotic–antimycotic (100 units/mL penicillin, 100 units/mL streptomycin, 250 ng mL^−1^ amphotericin B) (Gibco #15 240 062, Carlsbad, CA, US). Vero cells obtained from the Ministry of Food and Drug Safety (Osong, Republic of Korea) were maintained in Minimum Essential Medium (MEM) with Earle's Balanced Salts (HyClone #SH30024.01, Logan, UT) supplemented with 10% FBS (HyClone #SH30084.03) and 1% penicillin‐streptomycin (HyClone #SV300010). CFF‐16HBEge CFTR F508del cells (V470), a bronchial epithelial cell line harboring the ΔF508‐CFTR mutation, were obtained under the Material Transfer Agreement (MTA) from the Cystic Fibrosis Foundation and were maintained in Minimum Essential Medium (MEM) (Gibco #11095‐072, Carlsbad, CA) supplemented with 10% FBS and 1% penicillin/streptomycin (Gibco #15140‐122, Carlsbad, CA, US). The HEK293T‐ACE2‐TMPRSS2 cells were commercially purchased from Genecopoeia, Rockville, MD, USA, and maintained in culture medium in the presence of 1 µgmL^−1^ of puromycin and 100 µgmL^−1^ of Hygromycin B (USA #10 687 010; Invitrogen, Carlsbad, CA). For the generation of stable ACE2 overexpressing HeLa cells, hACE2 and P2A‐BSD encoding sequences were amplified from pcDNA3.1‐hACE2 (Addgene #145 033) and lentiCas9‐Blast (Addgene #52 962), respectively. The amplicons were cloned into the lentiviral pLV‐mCherry vector using the NEBuilder HiFi DNA Assembly Kit (NEB Biolabs). HeLa cells were transduced with lentiviral particles encoding hACE2‐P2A‐BSD in the presence of 8 µg/ mL^−1^ of polybrene (Merck‐Millipore, Burlington, MA, USA) for 24 h. Cells were maintained at a low density in the presence of 10 µg/ mL^−1^ of blasticidin for the selection.

All experiments using human nasal epithelial cells were approved by the Institutional Review Board of Yonsei University College of Medicine (4‐2021‐1671). Informed consent was obtained from all participants. The cells were isolated from surrounding tissues of nasal polyps obtained from patients with chronic rhinosinusitis and who had no clinical history of asthma, aspirin sensitivity, or cystic fibrosis; subsequently, the cells were cultured under air–liquid interface culture conditions, as previously described.^[^ ^48^ ^]^ Cells were grown at 37 °C in a 5% CO2 incubator.

### Plasmids, Cloning, siRNA, and Transfection

Plasmids encoding human pCMV‐ΔF508‐CFTR, pCMV‐WT‐CFTR (pCMVNot6.2), pcDNA3‐HA‐ARF1‐Q71L, pClneo‐HA‐Syntaxin5, pClneo‐Myc‐Sar1‐T39N, mammalian expressible extracellular‐tagged full‐length HA‐ΔF508‐CFTR, and HA‐WT‐CFTR have been described previously.^[^ ^4^ ^]^ The plasmids encoding C‐terminal DYK‐tagged human hTMED3 (gene ID: 23423), hTMED9 (gene ID: 54732), and hTMED10 (gene ID: 10972) were commercially purchased (GenScript, Piscataway, NJ, US). To generate the plasmids encoding human hTMED2, hTMED3, hTMED9, and hTMED10 without any tag, stop codons were inserted before the C‐terminal DYK tags of hTMEDs by site directed mutagenesis. Glutathione S‐transferase (GST) tagged TMED3 domain constructs were generated using seamless DNA assembly (NEB #E2621, Ipswich, MA, US) of three PCR fragments including signal sequences of TMED3 (SS [a.a. 1–27]) with a GST tag and the remaining domain (full‐length TMED3 [a.a. 28–217], GOLD [a.a. 28–119], GOLD+CC [a.a. 28–180], CC [a.a. 137–180], CC+TM [a.a. 28–201], and ΔC [a.a. 28–201]) into the bacterial expression vector pGEX‐4T‐1 (GE Healthcare, Chicago, IL, US). The mammalian expression plasmids for WT‐ and H723R‐pendrin were subcloned into pcDNA3.1(+), as described previously.^[^ ^5^ ^]^ The mammalian expressible pcDNA3.1‐spike‐C9 (Addgene #145 032) and pBOB‐CAG‐spike‐HA (Addgene #141 347) plasmids were commercially purchased. The mammalian expressible pcDNA3.1‐spike plasmid was generated by inserting a stop codon before the C9 tag from pcDNA3.1‐spike‐C9 plasmid. pcDNA3‐ORF3a‐HA plasmids were cloned from the cDNA of SARS‐CoV‐2 genomic RNA using the PCR‐based Gibson assembly method (NEB #E2621L)

ON‐TARGETplus human *TMED*‐specific, GRASP55‐specific, and control scrambled siRNAs were commercially purchased (SMARTpool siRNAs: h*TMED1*, gene ID 11018; h*TMED2*, gene ID 10959; h*TMED3*, gene ID 23423; h*TMED4*, gene ID 222068; h*TMED5*, gene ID 50999; h*TMED6*, gene ID 146456; h*TMED7*, gene ID 51014; h*TMED8*, gene ID 283578; h*TMED9*, gene ID 54732; h*TMED10*, gene ID 10972; hGORASP2, gene ID 26003; Lafayette, CO, US). Transfection of both plasmid DNA and siRNA into HEK293 or HeLa cells was performed using the TransIT‐X2 Dynamic Delivery System (#MIR6006, Mirus Bio LLC, Madison, WI, US), following the manufacturer's protocol.

### Chemical Reagents and Antibodies

Thapsigargin (Sigma‐Aldrich #T9033), 1‐deoxynojirimycin hydrochloride (Sigma‐Aldrich #D9305), kifunensine (Sigma‐Aldrich #K1140), swainsonine (Sigma‐Aldrich #S8195), Dynasore hydrate (Sigma‐Aldrich #D7693), bafilomycin A1 (Sigma‐Aldrich #B1793), MG132 (Selleckchem #S2619), and Ponceau S (Sigma‐Aldrich #P3504) were purchased commercially. CSTMP was custom‐synthesized by Cayman Chemical (Ann Arbor, MI, USA) (CAS registry number 1000672‐89‐8).

The following antibodies were acquired commercially: anti‐CFTR clone M3A7 (Millipore #05‐583, Billerica, MA, USA), anti‐HA (Cell Signaling Technology #2367, Danvers, MA, USA), anti‐Myc (Cell Signaling Technology #2276), anti‐Aldolase A (Abcam #ab78339, Cambridge, UK), anti‐Pendrin (Santa Cruz, #sc‐23779), anti‐IRE1*α* (Cell Signaling Technology #3294), anti‐phospho S724 IRE1*α* (Abcam #ab48187), anti‐TMED3 (Abcam #ab223175), anti‐TMED9 (Proteintech #21620‐1‐AP), anti‐TMED10 (Proteintech #15199‐1‐AP), anti‐TMED2 (Proteintech #11981‐1‐AP), anti‐DYK (GenScript #A00187), anti‐LC3 (Sigma #L8918), anti‐GFP (Santa Cruz #sc‐9996), anti‐DYKDDDDK (Cell Signaling Technology #2368), anti‐HA (Novus #NB600‐362), anti‐Furin (Invitrogen #PA1‐062), anti‐GM130 (BD Biosciences #610 899), anti‐Calnexin (Abcam #ab219644), anti‐GRASP55 (Abcam #ab74579) anti‐SARS‐CoV‐2 S1 (GeneTex #GTX135356), and anti‐SARS‐CoV‐2 S2 (GeneTex # GTX632604).

### Reverse‐Transcription (RT‐PCR) and Quantitative PCR Analysis (qPCR)

Forty‐eight hours after transfection with siRNAs, total RNA was extracted from HEK293 cells using the Tri‐RNA reagent (Favorgen Biotech Corp. #FATRR 001, Taiwan) according to the manufacturer's protocol. To reverse‐transcribe the RNA into cDNA, purified RNA samples were combined with the RNA to cDNA EcoDry premix (Takara Bio Inc. #639549, Shiga, Japan). Mixtures were incubated at 42 °C for 1 h, then at 70 °C for 10 min.

The Applied Biosystem StepOne System (Applied Biosystems, Forster City, CA, USA) was used to perform qPCR. The real‐time PCR reaction was measured by detecting the binding of fluorescent SYBR Green dye to double‐stranded DNA. For PCR amplification, the total reaction volume was adjusted to 20 µL with RNase‐free water after mixing with 100 ng cDNA, 2 µL primer sets, 10 µL 2× SYBR premix Ex Taq, and 0.4 µL 50× ROX reference dye (Takara #RR420L). Amplification was performed under the following cycling conditions: 95 °C for 15 min, followed by 40 cycles of 95 °C for 15 s, and 60 °C for 40 s. Analyses were performed in triplicate for each cDNA. Relative mRNA gene expression was normalized with the housekeeping gene *GAPDH*, and the *ΔC_t_* value was calculated as follows: *ΔC_t_* = *C_t_* (*GAPDH*) − *C_t_* (target gene). The average *ΔC_t_* was then subtracted from each experimental condition described to yield the *ΔΔC_t_* value. The fold‐changes in gene expression were calculated as 2^−^ *^ΔΔCt^* relative to control samples after normalization to *GAPDH*. The primer sequences used for qPCR analysis were: *GAPDH*, forward primer 5′‐AAT CCC ATC ACC ATC TTC CA‐3′, reverse primer 5′‐TGG ACT CCA CGA CGT ACT CA‐3′; h*TMED1*, forward primer 5′‐GAG TCC ATT GAG ACC ATG CG‐3′, reverse primer 5′‐CGT TGA CAG CTG ACC AGA AG‐3′; h*TMED2*, forward primer 5′‐GCT GTA AAG CAC GAA CAG GA‐3′, reverse primer 5′‐AGG ACC AAA GGA CCA CTC TG‐3′; h*TMED3*, forward primer 5′‐TGA TTG ACT CCC AGA CGC AT‐3′, reverse primer 5′‐GAC TGA AGC TGA CCA CGA AC‐3′; h*TMED4*, forward primer 5′‐TGC ATC TCG ACA TCC AGG TT‐3′, reverse primer 5′‐GGA AGC GCT CTT CAC GAT AC‐3′; h*TMED5*, forward primer 5′‐CCT TCC CTC GAT AGC GAC TT‐3′, reverse primer 5′‐GCC TTC TGG AGA GGC AAG AT‐3′; h*TMED6*, forward primer 5′‐GCC CAC CAG ACT GGA TAC TT‐3′, reverse primer 5′‐CAC CCT GGG AGG TGT CTA TG‐3′; h*TMED7*, forward primer 5′‐AGA ACC GAG TCA GTG CTC TT‐3′, reverse primer 5′‐GAG GGC TTC TCC TAC TGA CC‐3′; h*TMED8*, forward primer 5′‐TGC GAC CGA TGA CTA TGA CA‐3′, reverse primer 5′‐GAG CCT CTC TCC ACA TCT CC‐3′; h*TMED9*, forward primer 5′‐TGC GAC CGA TGA CTA TGA CA‐3′, reverse primer 5′‐GAG CCT CTC TCC ACA TCT CC‐3′; h*TMED10*, forward primer 5′‐CCT GAC CAA CTC GTG ATC CT‐3′, reverse primer 5′‐CGT TGG TAT CAC GCA TCT CC‐3′.

### Surface Biotinylation and Immunoblotting

For surface biotinylation, 48 h after transfection, HEK293 cells grown in 0.02 mgmL^−1^ poly‐D‐lysine (Sigma Aldrich #P6407) rinsed 6‐well plates (1 × 10^6^ cells/well) were incubated for 5 min at 4 °C and washed three times with chilled phosphate‐buffered saline (PBS). Transmembrane proteins in the plasma membrane of cultured cells were then biotinylated with 1 mL biotin solution (0.3 mg mL^−1^ Sulfo‐NHS‐SS‐Biotin [Thermo Pierce #21331, Waltham, MA, USA] in chilled PBS) for 30 min at 4 °C in the dark. Next, the cells were incubated with a quenching buffer containing 0.5% bovine serum albumin (BSA) in PBS for 10 min at 4 °C to remove excess biotin and were then washed three times with PBS. The surface‐biotinylated cells were then harvested in lysis buffer containing 20 mm Tris (pH 7.4), 150 mm NaCl, 1% (v/v) NP40, 10% glycerol, and 1 mm EDTA‐Na_2_ supplemented with protease inhibitor cocktail (Sigma‐Aldrich, Roche #0 469 3159001). Cell lysates were homogenized via sonication for 20 s (1‐second pulses) followed by centrifugation at 16 000 × *g* for 20 min at 4 °C. The resulting supernatants, containing 400 µg total protein, were incubated with 200 µL 10% streptavidin agarose (Thermo Pierce #20349). The streptavidin‐bound, biotinylated proteins were centrifuged and washed five times with lysis buffer. Biotinylated proteins were eluted in 20 µL 2× SDS sample buffer supplemented with 0.02 g mL^−1^ DL‐dithiothreitol (DTT) (Sigma‐Aldrich #43815) by vortex mixer (1000 rpm) at 37 °C for 30 min and separated by SDS‐polyacrylamide gel electrophoresis. Separated proteins were electro‐transferred to a nitrocellulose membrane (GE Healthcare, Amersham #10600004, Chicago, IL, USA) and blotted with the appropriate primary and secondary antibodies (in 5% skim milk). Protein bands were detected by enhanced chemiluminescence (GE Healthcare, Amersham #RPN2105); and the densities of each protein band were quantified using imaging software (Multi Gauge ver. 3.0; Fujifilm, Tokyo, Japan).

Digestion of N‐glycosylated proteins by PNGase F (New England Biolabs, #P0704L) and Endo H (New England Biolabs, #P0702L) was performed with slight modifications to the manufacturer's instructions.^[^ ^4^ ^]^ Specifically, 50‐µg protein samples were first denatured by adding 0.5% SDS and 40 mm DTT and subsequently incubated at 37 °C for 10 min. The denatured protein samples were then treated with PNGase F (500 U/reaction, with 1% NP40) in a sodium phosphate‐containing solution (50 mm, pH 7.5) or treated with Endo H (1000 U/reaction) in a sodium acetate‐containing solution (50 mm, pH 6.0) at 37 °C for 2 h.

### Immunoprecipitation and Pull‐Down Assay

For co‐immunoprecipitation, 500 µg protein lysates were diluted to a final volume of 500 µL in lysis buffer (20 mm Tris [pH 7.4], 200 mm NaCl, 1% NP40 [v/v], 1 mm EDTA‐Na_2_ supplemented with protease inhibitor cocktail) and pre‐cleared by incubating with normal IgG at 4 °C for 30 min and with control agarose resin (crosslinked 4% bead agarose) at 4 °C for 2 h. The mixtures were then centrifuged for 10 min at 3000 rpm, and the protein lysates were collected from the supernatant. The pre‐cleared protein lysates were then incubated with appropriate antibodies overnight at 4 °C. To precipitate proteins of interest, immune complexes were collected by binding to pre‐equilibrated 10% protein A/G resin (Thermo Pierce #20421) in lysis buffer at 4 °C for 6 h, then washed five times with lysis buffer prior to elution with 2× SDS‐PAGE sample buffer, electrophoresis, and immunoblotting.

The pull‐down assay was performed as previously described.^[^ ^4^ ^]^ All recombinant fusion proteins were produced in the BL‐21 (DE3) *Escherichia coli* strain. Synthesis of glutathione‐S‐transferase (GST) fusion proteins was induced with 0.5 mm isopropyl *β*‐D‐1‐thiogalactopyranoside (IPTG) at 30 °C. Recombinant proteins were subsequently purified with glutathione Sepharose 4B resin (GE Healthcare #17 075601) according to the manufacturer's instructions. 100 µg CFTR proteins expressed in HEK293 cells were mixed with 50 µg of GST‐fusion recombinant proteins bound to glutathione Sepharose and incubated overnight at 4 °C. After vigorous washing with lysis buffer five times, bead‐bound proteins were eluted with 2× SDS sample buffer and then subjected to immunoblotting. GST‐fusion recombinant proteins were detected using Ponceau S staining.

### Immunocytochemistry, Confocal Microscopy, and Morphometric Analysis

Immunofluorescence staining was performed as previously described, with slight modifications.^[^ ^49^ ^]^ For immunocytochemistry, HeLa cells were cultured on 18‐mm round coverslips and fixed with 4% paraformaldehyde (diluted in PBS) for 7 min at room temperature. Then, fixed cells were permeabilized with 0.2% Triton X‐100 in PBS at room temperature for 5 min, before undergoing three washes with PBS and incubation with blocking solution (1% bovine serum albumin [BSA] and 5% horse serum in PBS) at room temperature to block non‐specific binding sites. After blocking, cells were first stained with primary antibodies and then with secondary fluorophore‐conjugated antibodies. The following antibodies were used for staining: anti‐CFTR clone M3A7 (Millipore #05‐583, Billerica, MA, USA), anti‐TMED3 (Abcam # ab223175), anti‐DYKDDDDK (Cell Signaling Technology #2368), anti‐HA (Novus #NB600‐362), anti‐Furin (Invitrogen #PA1‐062), anti‐GM130 (BD Biosciences #610 899), anti‐Calnexin (Abcam #ab219644), anti‐SARS‐CoV‐2 S1 (GeneTex # GTX135356), and anti‐SARS‐CoV‐2 S2 (GeneTex # GTX632604). Cells on coverslips were mounted to glass slides with fluorescence mounting medium (Agilent Dako #S3025, Santa Clara, CA, USA). Fluorescent images were captured using a laser scanning confocal microscope (LSM 980; Carl Zeiss, Berlin, Germany) with a 63× 1.4 numerical aperture oil objective lens.

Morphometric analysis of the captured confocal images was performed using MetaMorph microscopy analysis software (version 7.1; Molecular Devices, Sunnyvale, USA) as previously described.^[^ ^49^ ^]^ Briefly, for image quantification under each condition, 24‐bit confocal images including red, green, and blue components were converted into three 8‐bit mono‐channel images. To quantify Spike intensity, pixels above a threshold level of 60 were defined as Spike positive. The intensity profile of each individual cell was presented as the standard deviation around the mean of the average intensity value for the entire region.

The degree of colocalization between CFTR and TMED3 was quantified based on Manders’ colocalization coefficients (MCC);^[^ ^50^ ^]^ using the colocalization module of the ZEN 2012 software (black edition; Carl Zeiss, Berlin, Germany). For MCC measurement under each condition, pixels above the fluorescence threshold level of 50 for both channels (red and green) were defined as overlapping signals. Then, the average MCC obtained from the regions of interest was calculated. For the two target proteins, represented as A and B, two different MCC values were calculated as:

(1) $${\mathrm{MCC}}_{A\;\mathrm{colocalized}\;\mathrm{with}\;B/\text{Total}\;A}=\frac{\sum_{i}A_{i,\mathrm{colocal}}}{\sum_{i}A_{i}}$$

(2) $${\mathrm{MCC}}_{B\;\mathrm{colocalized}\;\mathrm{with}\;A/\text{Total}\;B}=\frac{\sum_{i}B_{i,\mathrm{colocal}}}{\sum_{i}B_{i}}$$

### Measurements of Cl^−^ Channel Activity and Short‐Circuit Currents (*I*_sc_)

Whole‐cell recordings were performed on CFTR transfected HEK293 cells as described previously.^[^ ^4^ ^]^ Cells were transferred into a bath mounted on the stage of an inverted microscope (Ti2, Nikon) and the whole‐cell patch was achieved by rupturing the membrane after giga‐ohm sealing. The bath solution was perfused at 5 mL min^−1^. The voltage and current recordings were performed at room temperature (22–25 °C). Patch pipettes with a resistance of 2–4 MΩ were connected to the head stage of a patch clamp amplifier (Axopatch‐200B, Molecular Devices, Sunnyvale, CA, USA). The bath solution contained (in millimolar) 140 N‐methyl‐D‐glucamine chloride (NMDG‐Cl), 1 CaCl_2_, 1 MgCl_2_, 10 glucose, and 10 HEPES (pH 7.4). The pipette solution contained (in millimolar) 140 N‐methyl‐D‐glucamine chloride, 5 EGTA, 1 MgCl_2_, 3 MgATP, and 10 HEPES (pH 7.2). To determine the current–voltage (I/V) relationship, the voltage clamp mode and I/V curve were obtained by applying ramp pulses ranging from −100 to 100 mV (0.8 mV ms^−1^, holding potential 0 mV). CFTR currents were activated by cAMP (5 µm forskolin and 100 µm 3‐isobutyl‐1‐methylxanthine [IBMX]). The current generated by the CFTRs was confirmed by applying the CFTR inhibitor CFTR_inh_‐172 (10 µm). To acquire data and apply command pulses, pClamp 10.2 and Digidata 1550B (Molecular Devices) were used. Voltage and current traces were stored and analyzed using pClamp 10.2 and Origin 8.0 (OriginLab Corp., Northampton, MA, USA). The currents were filtered at 5 kHz and sampled at 1 kHz. All data were normalized to the whole‐cell capacitance (pF).

### Measurement of Cl^−^/HCO_3_^−^ Exchange Activity

Measurements of pH_i_ in PANC‐1 cells were performed with the pH‐sensitive fluorescent probe 2',7'‐bis‐(2‐carboxyethyl)‐5‐(and‐6)‐carboxyfluorescein (BCECF) in accordance with the previously reported protocols.^[^ ^5^ ^]^ Briefly, cells were incubated with 2 µm BCECF acetoxy‐methylester for 5 min and then perfused with a HCO_3_^−^‐buffered solution (containing [in mmol L^−1^]: 120 NaCl, 5 KCl, 1 MgCl_2_, 1 CaCl_2_, 10 D‐glucose, 5 HEPES, and 25 NaHCO_3_, pH 7.4). BCECF fluorescence was recorded at excitation wavelengths of 490 and 440 nm at a resolution of 2/s on a recording setup (Delta Ram; PTI Inc., Edison, New Jersey, USA). The Cl^−^/HCO_3_^−^ exchange activity was estimated from the initial rate of pH_i_ increase due to Cl^−^ removal from the HCO_3_^−^‐containing buffer (25 mm HCO_3_^−^ with 5% CO_2_). The pH_i_ calibration was performed with standard pH solutions containing 150 mm KCl and 5 µm nigericin. The intrinsic buffer capacity (*β*_i_) was calculated by measuring ΔpH_i_ in response to 5–40 mm NH_4_Cl pulses in Na^+^‐free solutions. Because the *β*_i_ values were not significantly affected by transfection with the plasmids encoding for WT‐pendrin or H723R‐pendrin, the Clˉ/HCO_3_ˉ exchange activities were expressed as ΔpH unit/min without compensating for the buffer capacity.

### Virus Propagation, Quantification, and Infection of SARS‐CoV‐2

A clinically isolated SARS‐CoV‐2 KUMC‐2 strain (GISAID accession#: EPI_ISL_413 018) was provided by Prof. MS Park at Korea University. For the propagation of viruses, Vero cells were infected by SARS‐CoV‐2 with 0.01 multiplicity of infection (MOI) and then culture supernatant was harvested at 24–48 h post‐infection. The harvested supernatant was filtered and stocked at −80 ℃. For plaque formation assay, one of the stock vials was used to infect a Vero cell, which was then incubated with culture media containing 1% low melting temperature agarose (Lonza #50101, Rockland, ME) for 48 h. Viral plaques were visualized with Neutral Red Solution (Sigma #2889, Sigma‐Aldrich, St. Louis, MO) for an additional 24 h and were counted to determine the plaque‐forming unit of the viral stock. For viral infection assays, target cells were incubated with a virus of 0.01 multiplicity of infection (MOI) for 1 h, then replaced with media complemented with 2% FBS until subsequent experimentation. To obtain lentiviral particles, pLKO.1 shRNA plasmids (1 µg) were co‐transfected with psPAX2 packaging plasmids (750 ng) and pMD2.G envelope plasmids (250 ng) into HEK293 cells. To establish stable TMED knockdown cell lines, Vero cells were infected with lentiviral particles purified from supernatants of HEK293 cells expressing TMED3, TMED9, TMED10, and TMED2 shRNAs. After 24 h, the supernatant was replaced with a complete medium supplemented with 5 µg mL^−1^ puromycin. Following puromycin screening, transduced cells were harvested for qPCR analysis. The target sequences of shRNAs were: *TMED2* (gene ID: 10959) 5′‐GAG CCA TCA ACG ACA ACA CAA‐3; *TMED3* (gene ID: 23423) 5′‐CTC TCA CAA GAC CGT CTA CTT‐3′; *TMED9* (gene ID: 54732) 5′‐GCT GCT AAA GAC AAG TTG AGT‐3′; *TMED10* (gene ID: 10972) 5′‐CAA CAA ACA CTC GGG TCC TAT‐3′. All experiments with infectious viruses were approved by the Institutional Biosafety Committee of Yonsei University Health System (IBC 2020‐003) and conducted in the Biosafety Level 3 facility at Yonsei University College of Medicine.

### Quantification of Viruses

For the quantification of the SARS‐CoV‐2 virus secretion, viral RNA in cell culture supernatant was extracted using a QIAamp Viral RNA Mini kit (QIAGEN #52906, Valencia, CA), as per the manufacturer's instructions. Quantitative PCR was performed with a Luna Universal One‐Step RT‐qPCR Kit (NEW ENGLAND BioLabs Inc., Ipswich, Massachusetts, USA) using the ABI Prism 7000 detection system (Applied Biosystems, Waltham, MA, USA). For amplification, 2 µL of extracted RNA was added to a mixture of 10 µL of 2× Luna Universal One‐Step Reaction Mix, 0.5 µL of 10 µm primers, 0.25 µL of 10 µm probe and 1 µL of 20× Luna WarmStart RT Enzyme Mix adjusted to a total reaction volume of 20 µL with RNase‐free water. Amplification was carried out using the optimal thermocycling condition outlined in the manufacturer's protocol for 40 cycles. The primer‐probe sets, targeting nsp14,^[^ ^51^ ^]^ were used: forward primer 5′‐TGGGGYTTTACRGGTAACCT‐3′, reverse primer 5′‐AACRCGCTTAACAAAGCACTC‐3′, probe 5′‐56‐FAM‐ FAM‐TAGTTGTGA/ZEN/TGCWATCATGACTAG‐3IABkFQ‐3′. The standard curve was generated by conducting qRT‐PCR using serially diluted virus stock. Then, Ct values were converted into viral titer (PFU/mL).

### Statistical Data Analysis

The results of multiple experiments are presented as the mean ± standard error of the mean (SEM). Statistical analysis was performed using a two‐tailed Student's *t*‐test or one‐way analysis of variance followed by Tukey's multiple comparison test as appropriate, using GraphPad Prism 8 (GraphPad Software, Inc., La Jolla, CA, USA). *p* < 0.05 was considered statistically significant.

## Conflict of Interest

The authors declare no conflict of interest.

## Author Contributions

H.P., S.K.S., J.‐R.S., S.J.H., and Y.J.K. contributed equally to this work. H.P. and Y.J.K. designed and carried out the molecular experiments on CFTR. S.K.S. designed and carried out the molecular experiments with SARS‐CoV‐2 Spike. J.S. designed and carried out the molecular experiments on pendrin. S.J.H. performed the experiments with infectious virus particles. D.H.S. and D.G.J. performed functional assays for CFTR and pendrin, respectively. S.H.N. performed immunocytochemistry. P.G.P. and S.H.K. assisted with the experiments involving infectious viruses. M.H.S. and J.Y.C. performed experiments with HNE cells. Y.I. aided with the experiments using N‐glycans. C.K. and J.M.L. designed and conceived experiments pertaining to pendrin and SARS‐CoV‐2, respectively. M.G.L. designed and conceived all experiments and wrote and edited the manuscript.

## Supporting information

Supporting Information

## Data Availability

The data that support the findings of this study are available from the corresponding author upon reasonable request.
