# Supplementary material for: TMED3 Complex Mediates ER Stress‐Associated Secretion of CFTR, Pendrin, and SARS‐CoV‐2 Spike
Source: Adv Sci (Weinh). 2022 Jun 24;9(24):2105320. doi: 10.1002/advs.202105320 (PMC9350134; doi:10.1002/advs.202105320)
Supplement: Supplementary file 1 — Supporting Information [file ADVS-9-2105320-s001.pdf]

ADVANCED SCIENCE
Open Access

Supporting Information

for *Adv. Sci.*, DOI 10.1002/advs.202105320

# TMED3 Complex Mediates ER Stress-Associated Secretion of CFTR, Pendrin, and SARS-CoV-2 Spike

*Hak Park, Soo Kyung Seo, Ju-Ri Sim, Su Jin Hwang, Ye Jin Kim, Dong Hoon Shin, Dong Geon Jang, Shin Hye Noh, Pil-Gu Park, Si Hwan Ko, Mi Hwa Shin, Jae Young Choi, Yukishige Ito, Chung-Min Kang*, Jae Myun Lee* and Min Goo Lee**

# Supporting Information for

## TMED3 complex mediates ER stress-associated secretion of CFTR, pendrin, and SARS-CoV-2 Spike

**Hak Park[1,2,†], Soo Kyung Seo[1,†], Ju-Ri Sim[1,†], Su Jin Hwang[3,8,†], Ye Jin Kim[1,†], Dong Hoon Shin[1], Dong Geon Jang[1], Shin Hye Noh[1], Pil-Gu Park[3], Si Hwan Ko[3], Mi Hwa Shin[4], Jae Young Choi[4], Yukishige Ito[5,6], Chung-Min Kang[7,*], Jae Myun Lee[3,8,*], Min Goo Lee[1,8,*]**

[*] Corresponding authors:

1) Min Goo Lee, E-mail: mlee@yuhs.ac

2) Jae Myun Lee, E-mail: jaemyun@yuhs.ac

3) Chung-Min Kang, E-mail: KANGCM@yuhs.ac

**This PDF file includes:**

Table S1
Figs. S1 to S11

**Supplementary Table 1. Protein and mRNA expression of UPS molecular players in the respiratory tissues.**

| Tissues | IRE1α (ERN1) | Grasp55 (GORASP2) | TMED2 | TMED3 | TMED9 | TMED10 |
|---|---|---|---|---|---|---|
| Nasopharynx (Respiratory epithelial cells) | Medium | High | High | Low | High | High |
| Bronchus (Respiratory epithelial cells) | High | High | Medium | Medium | High | Medium |
| Lung (Alveolar cells) | Medium | Low | Medium | ND | Low | Low[1] |
| Lung (Endothelial cells) | ND | NA | NA | NA | NA | ND |
| Lung (Macrophages) | Medium | Low | Medium | ND | Medium | Low |

Protein expression data are taken from the Human Protein Atlas (https://www.proteinatlas.org/).
[1]Alveolar cell type I: not detected, Alveolar cell type II: Low
ND: Not detected
NA: Not available

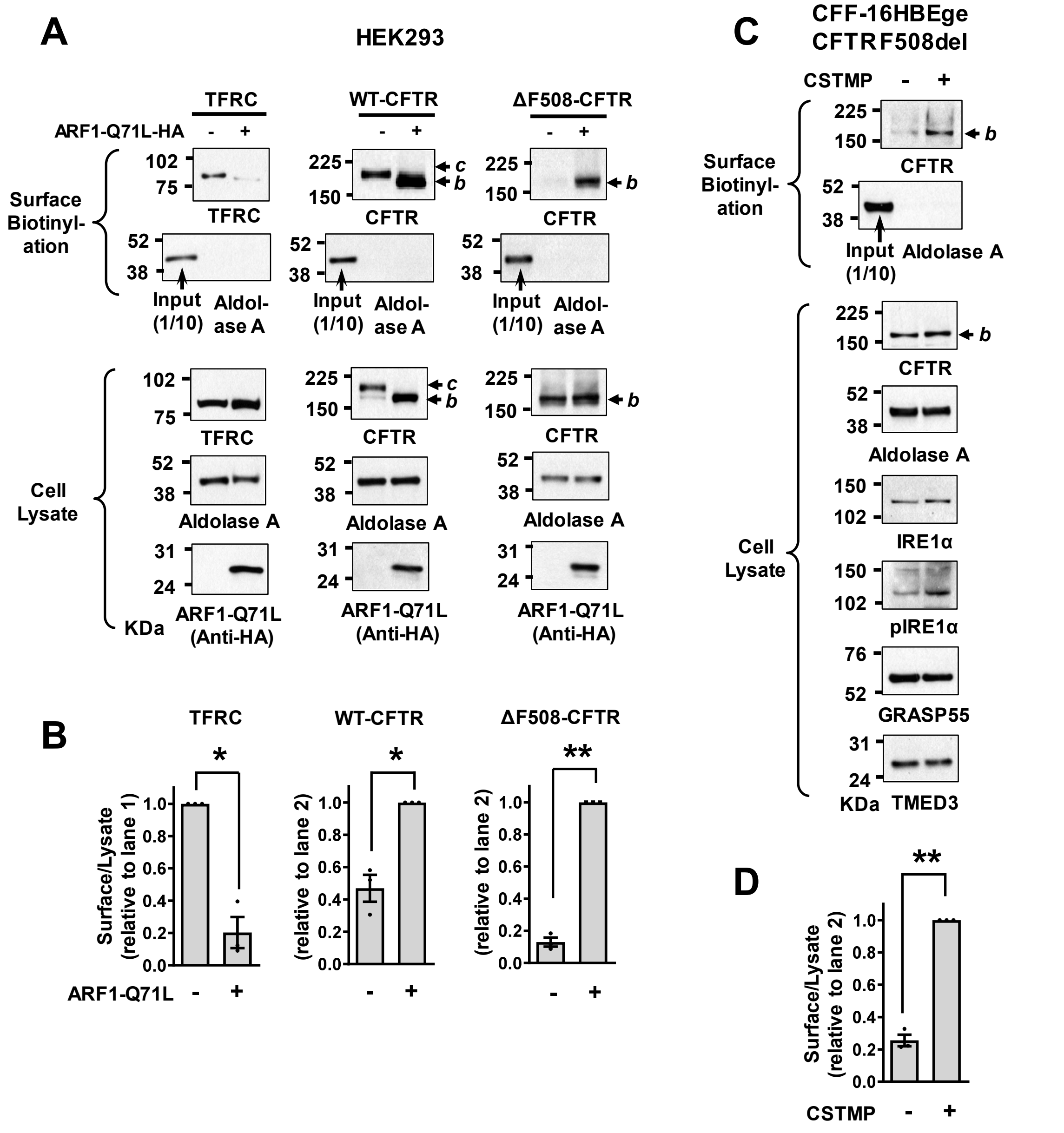


**Figure S1. CFTR, but not the transferrin receptor (TFRC), undergoes a UPS pathway. (A and B)** Effects of ER-to-Golgi blockade on the protein secretion of transmembrane proteins, CFTR and transferrin receptor (TFRC), were analyzed. The cell surface biotinylation assay was performed in HEK293 cells transfected with plasmids encoding TFRC, WT- or ΔF508-CFTR. ARF1-Q71L was co-expressed in some cells to induce ER-to-Golgi blockade. Representative blots of surface biotinylation are shown in (A). Quantifications of multiple experiments are summarized in (B, n = 3). **(C and D)** The cell surface biotinylation assay was performed in CFF-16HBEge CFTR F508del cells, a bronchial epithelial cell line harboring the ΔF508-CFTR mutation, with or without the IRE1α kinase activator CSTMP (10 μM, 24 h). CSTMP induces the UPS ΔF508-CFTR. Representative blots of surface biotinylation are shown in (C). Quantifications of multiple experiments are summarized in (D, n = 3). Bar graph data are shown as mean ± SEM. $^{**}P < 0.01$. Data were analyzed using a two-tailed Student's *t*-test. ***b***, ER core-glycosylated CFTR.

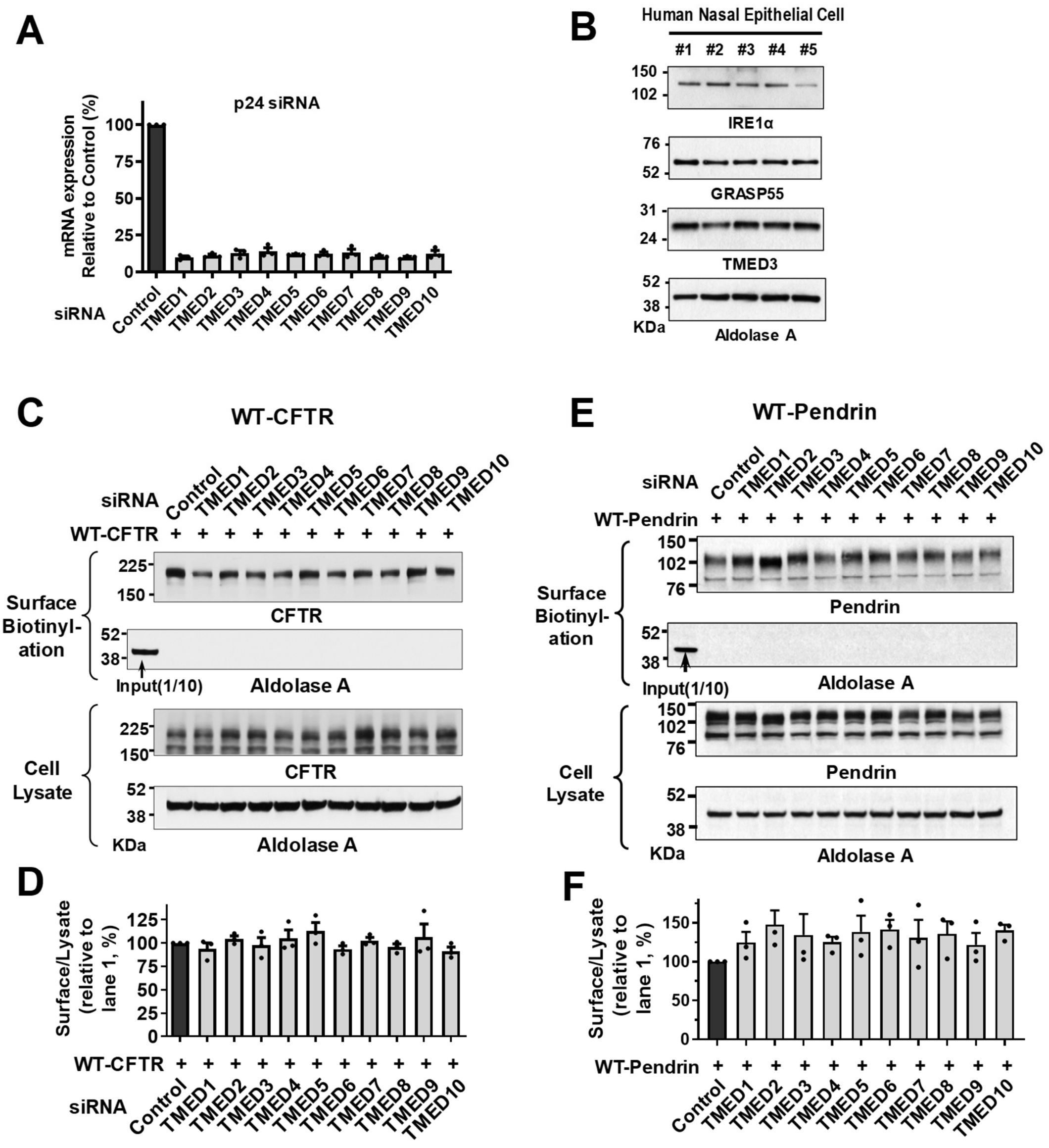


**Figure S2. TMEDs are not involved in the conventional protein secretion of wild-type CFTR and pendrin. (A)** mRNA samples were extracted from HEK293 cells after transfection with control (scrambled) or siRNA against each *TMED* gene (100 nM, 48 h). Data are shown as mean ± SEM (n = 3). **(B)** Expressions of IRE1α, GRASP55, and TMED3 were analyzed in the primarily cultured human nasal epithelial cells obtained from five different individuals. **(C and D)** Effects of individual *TMED* gene silencing on the conventional protein secretion of wild-type (WT) CFTR. The cell surface biotinylation assay was performed in HEK293 cells transfected with control (scrambled) or *TMED*-specific siRNAs together with plasmids encoding CFTR. Representative blots of surface biotinylation assays are shown in (C). Quantifications of multiple experiments are summarized in (D, n = 3). Silencing of TMEDs did not affect conventional secretion of WT-CFTR. Data are shown as mean ± SEM. **(E and F)** Effects of individual *TMED* gene silencing on the conventional protein secretion of WT-pendrin. The cell surface biotinylation assay was performed in cells transfected with plasmids encoding pendrin. Representative blots are shown in (E). Quantifications of multiple experiments are summarized in (F, n = 3). Silencing of TMEDs did not affect conventional secretion of WT-pendrin. Bar graph data are shown as mean ± SEM.

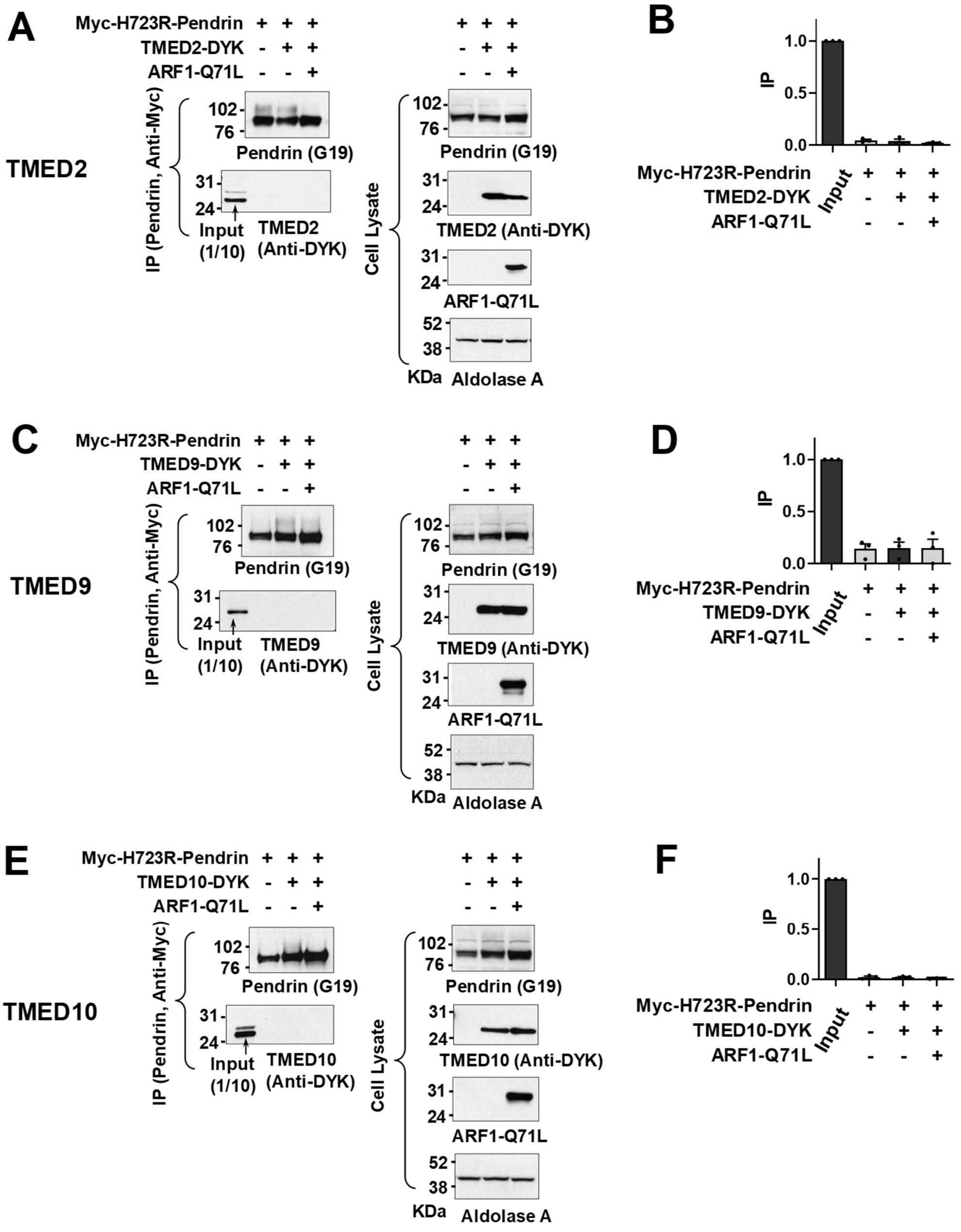


**Figure S3. TMED2, TMED9, and TMED10 do not associate with p.H723R-pendrin.** Co-immunoprecipitation (co-IP) experiments were performed with anti-Myc antibodies in PANC-1 cells transfected with plasmids encoding Myc-tagged p.H723R-pendrin and DYK-tagged TMED proteins. The co-IP results of TMED3 are shown in Fig. 2, C and D. ARF1-Q71L was co-expressed in some cells to induce UPS. Protein samples were blotted with antibodies against each labeled protein. The HA-tagged ARF1-Q71L was blotted with anti-HA antibodies. **(A and B)** Co-IP with DYK-tagged TMED2. Summarized results of multiple experiments are presented in (B, n = 3). **(C and D)** Co-IP with DYK-tagged TMED9. Summarized results of multiple experiments are presented in (D, n = 3). **(E and F)** Co-IP with DYK-tagged TMED10. Summarized results of multiple experiments are presented in (F, n = 3). Bar graph data are shown as mean ± SEM.

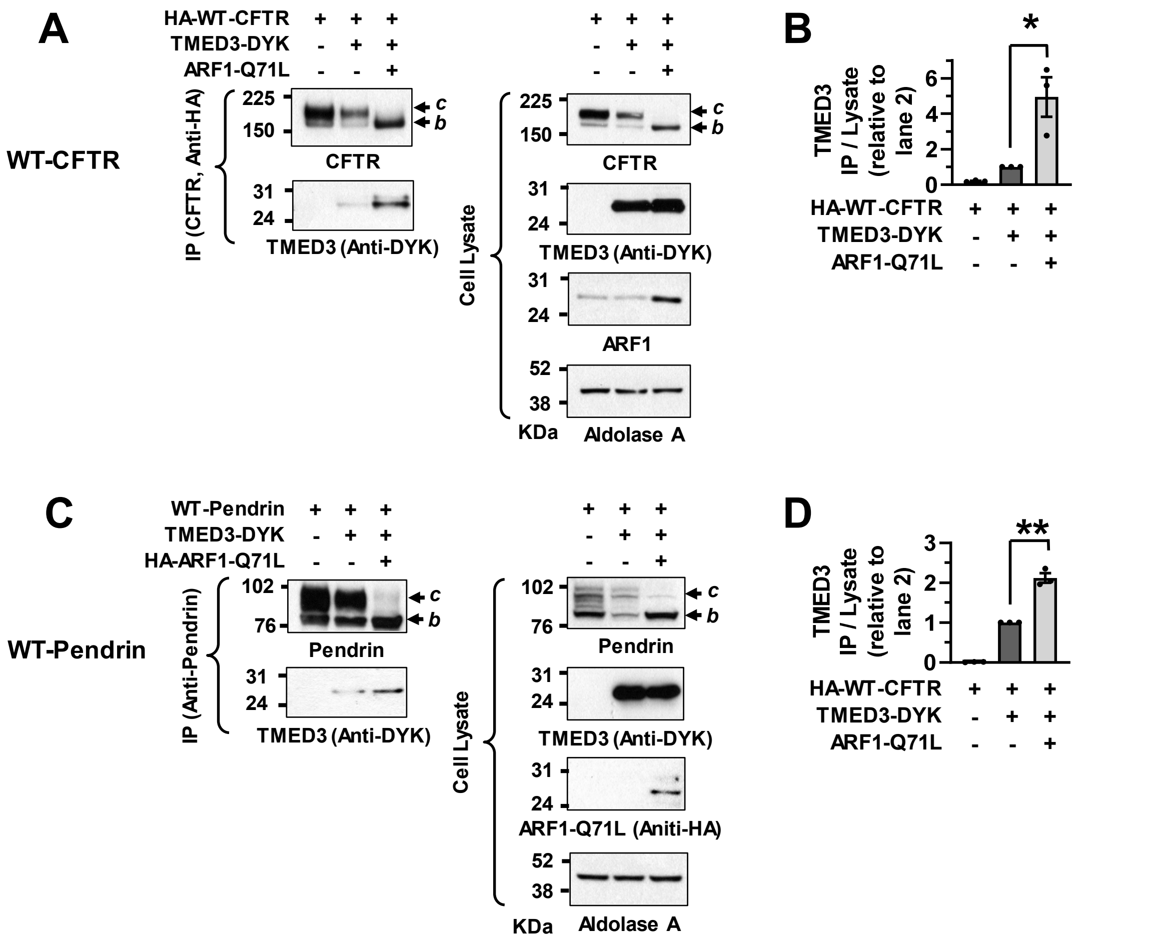


**Figure S4. Interactions between TMED3 and wild-type (WT) CFTR and with WT-Pendrin are augmented by ARF1-Q71L. (A and B)** Co-immunoprecipitation (co-IP) experiments of WT-CFTR and TMED3 were performed with anti-HA antibodies in HEK293 cells transfected with plasmids encoding HA-tagged WT-CFTR solely or those with TMED3 (TMED3-DYK). ARF1-Q71L was co-expressed in some cells to induce UPS. WT-CFTR interacted slightly with TMED3 and the interaction was increased by ARF1-Q71L. Protein samples were blotted with antibodies against each labelled protein (A). Summarized results of multiple experiments are presented in (B, n=3). **(C and D)** Co-IP experiments of WT-Pendrin and TMED3 were performed with anti-Pendrin antibodies (R2) in PANC-1 cells transfected with plasmids encoding WT-Pendrin alone or those with TMED3 (TMED3-DYK). Interaction between WT-Pendrin and TMED3 was enhanced by ARF1-Q71L. Protein samples were blotted with antibodies against each labelled protein (C). Summarized results of multiple experiments are presented in (D, n=3). Bar graph data are shown as mean ± SEM. **P* < 0.05, ***P* < 0.01: difference from lane 2. Data were analyzed using one-way analysis of variance, followed by Tukey's multiple comparison test.

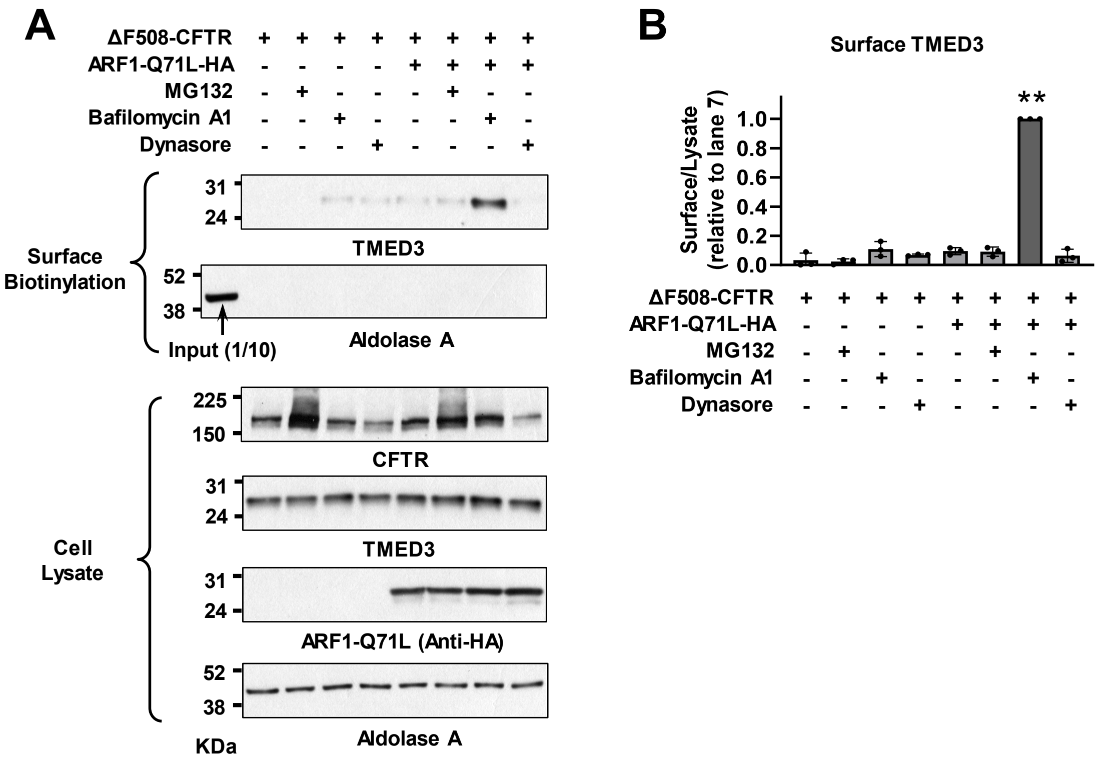


**Figure S5. Inhibition of lysosomal function triggers cell surface trafficking of TMED3 under the ARF1-Q71L-induced UPS condition.** Effects of individual intracellular organelle inhibitors on the plasma membrane transport of TMED3. The cell surface biotinylation assay was performed in HEK293 cells transfected with plasmids encoding ΔF508-CFTR. ARF1-Q71L was co-expressed in some cells to induce UPS. Some cells were treated with MG132 (10 µM, 4 h), bafilomycin A1 (20 nM, 20 h), and dynasore (80 µM,12 h) to inhibit proteasomal degradation, lysosomal V-type $H^+$-ATPase, and vesicular endocytosis and recycling, respectively. Representative blots of surface biotinylation assays are shown in (A). Quantifications of multiple experiments are summarized in (B, n = 3). Under the ARF1-Q71L induced ER-to-Golgi blockade conditions, the cell surface expression of TMED3 was increased by bafilomycin A1. Data are shown as mean ± SEM.

TMED3-Pendrin, Pull-down

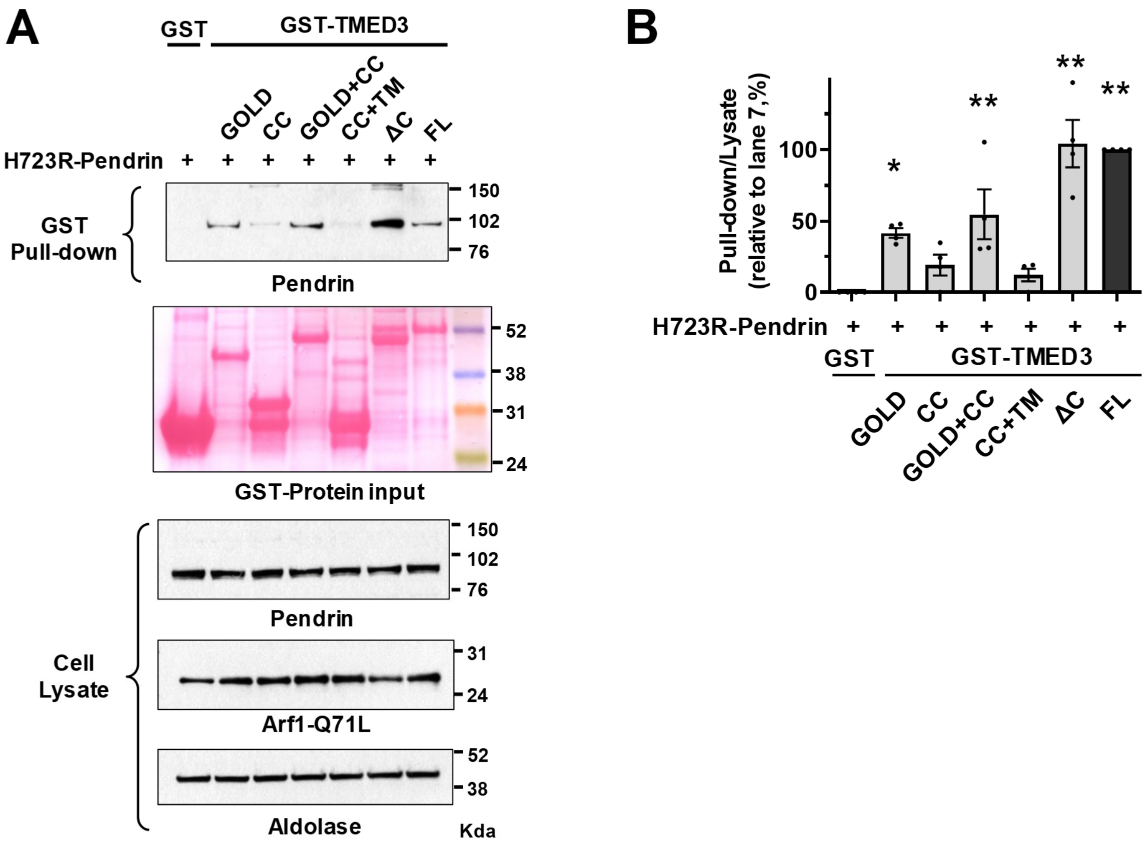


**Figure S6. The GOLD domain of TMED3 associates with p.H723R-pendrin.** Pull-down assays were performed with domain-specific GST-fused TMED3 proteins (100 µg) and cell lysates extracted from PANC-1 cells expressing p.H723R-pendrin (400 µg). Schematic diagrams of GST-tagged TMED3 proteins are depicted in Figure 5A. The input GST-fused recombinant proteins were visualized using Ponceau S staining and pendrin was immunoblotted with anti-pendrin antibodies. Representative pull-down assays are shown in (A) and the results of multiple experiments are summarized in (B, n = 4). Bar graph data are shown as mean ± SEM.

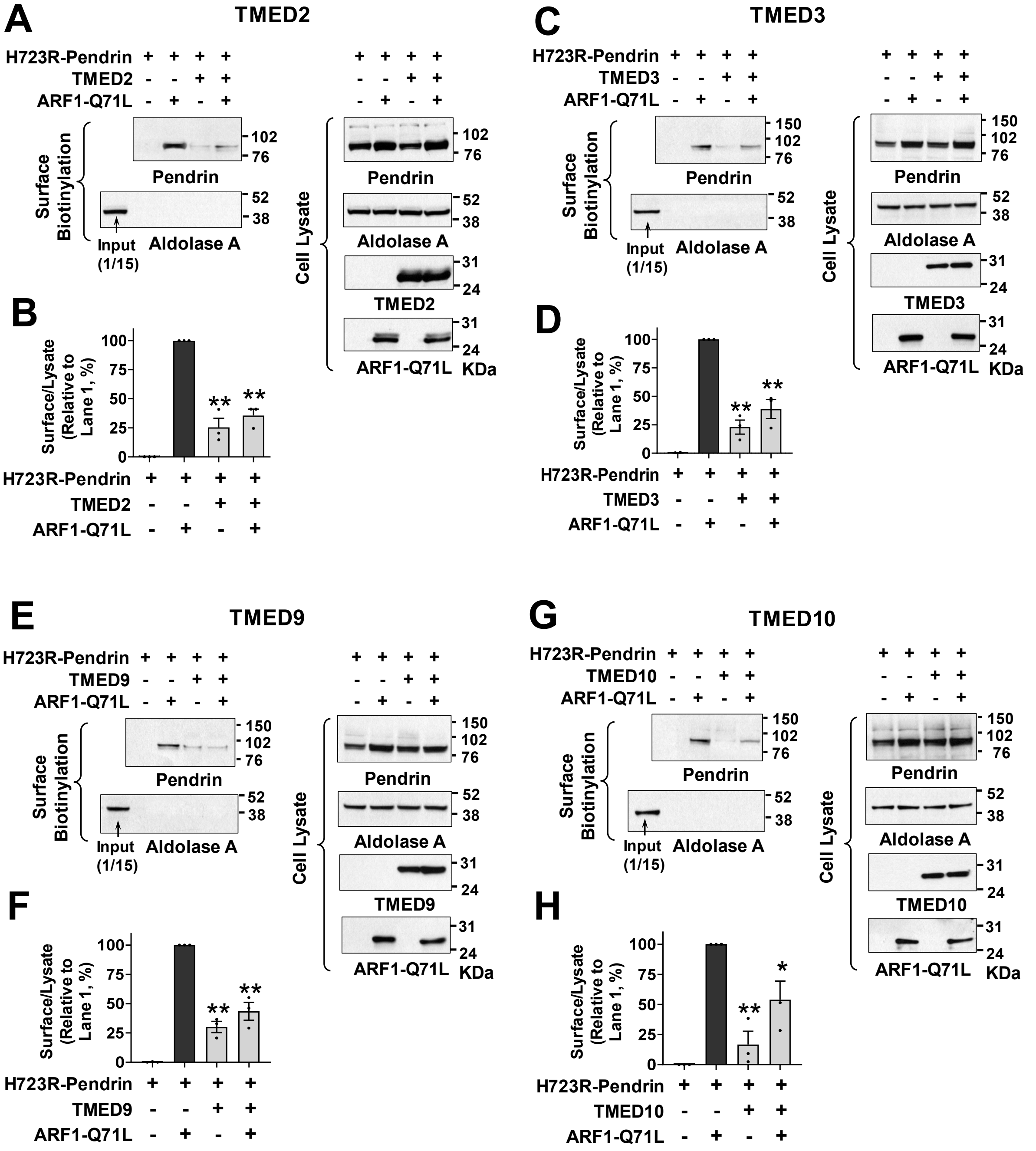


**Figure S7. Single overexpressions of TMED2, 3, 9, and 10 inhibit UPS of pendrin.** The effects of single overexpressions of TMED2 (A and B), TMED3 (C and D), TMED9 (E and F), and TMED10 (G and H) on the ARF1-Q71L-induced UPS of p.H723R-pendrin were examined using the surface biotinylation assays. Representative blots are shown in (A, C, E and G), respectively. The results of multiple experiments are summarized in (B, D, F and H; n = 3), respectively. Bar graph data are shown as mean ± SEM. *$P < 0.05$, **$P < 0.01$. difference from lane 2. Data were analyzed using one-way analysis of variance, followed by Tukey’s multiple comparison test.

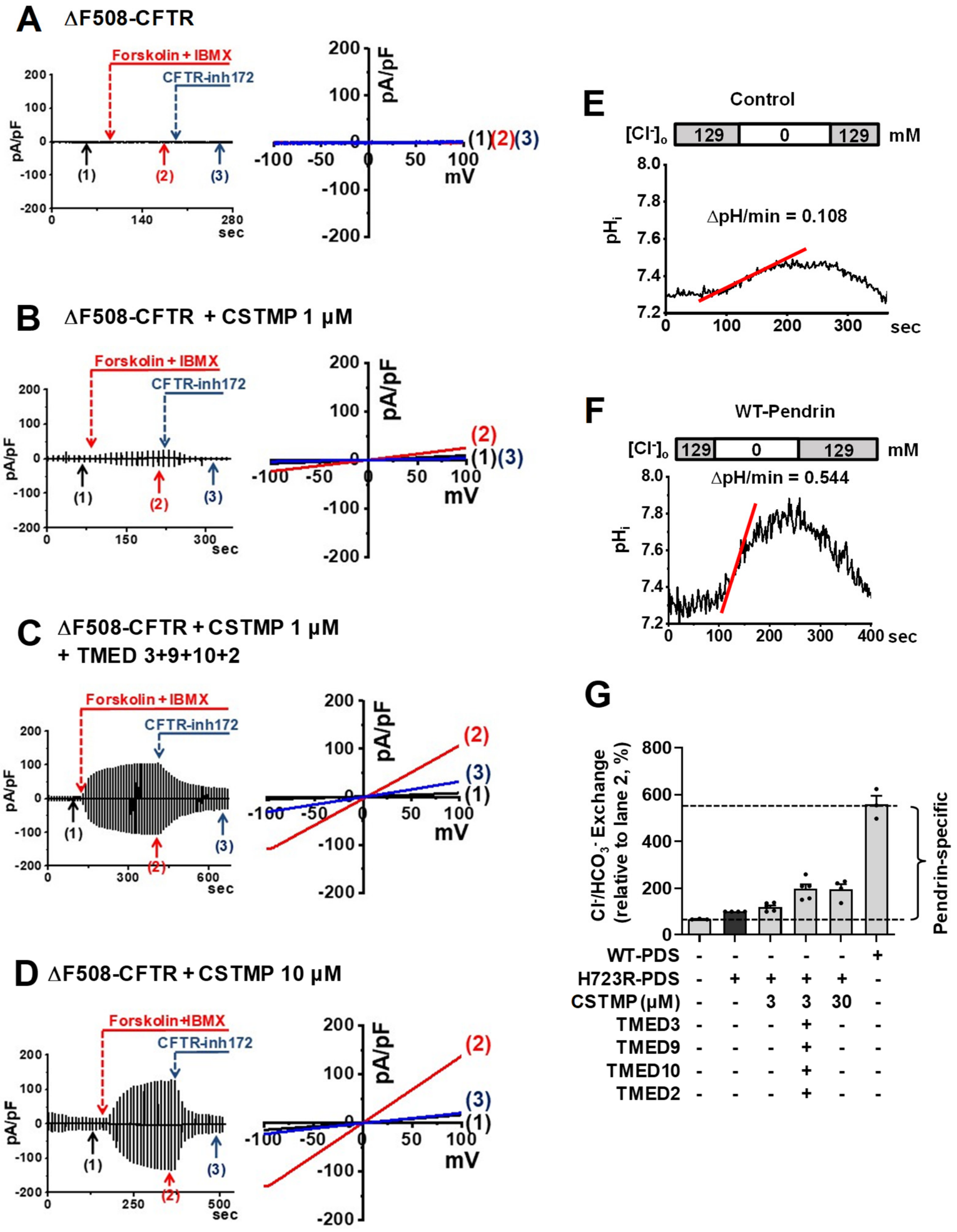


**Figure S8. Measurements of ion transporting activities of CFTR and pendrin. (A-D)** Current/voltage (I/V) relationship for the whole-cell current measurements in Figure 7, A-D. To determine the I/V relationship, the clamp mode was shifted to the voltage clamp mode and the I/V curve was obtained by applying ramp pulses ranging from −100 mV to 100 mV. Each reversal potential measurement is marked with an arrow (1, 2, 3). **(E-G)** Measurements of pendrin-mediated $Cl^-/HCO_3^-$ exchange activity. The intracellular pH ($pH_i$) measurements in PANC-1 cells were performed with the pH-sensitive fluorescent probe 2',7'-bis-(2-carboxyethyl)-5-(and-6)-carboxyfluorescein (BCECF). The $Cl^-_o/HCO_3^-{}_i$ exchange activities were estimated from the initial rate of $pH_i$ increase due to $Cl^-$ removal from the $HCO_3^-$-containing buffer (25 mM $HCO_3^-$ with 5% $CO_2$). The pendrin-specific anion exchange activity was measured by subtracting the basal activity in control cells (E) from the anion exchange activity of wild-type (WT) pendrin expressing cells (F). Quantifications of multiple experiments are summarized in (G, n = 3–5). Bar graph data are shown as mean ± SEM.

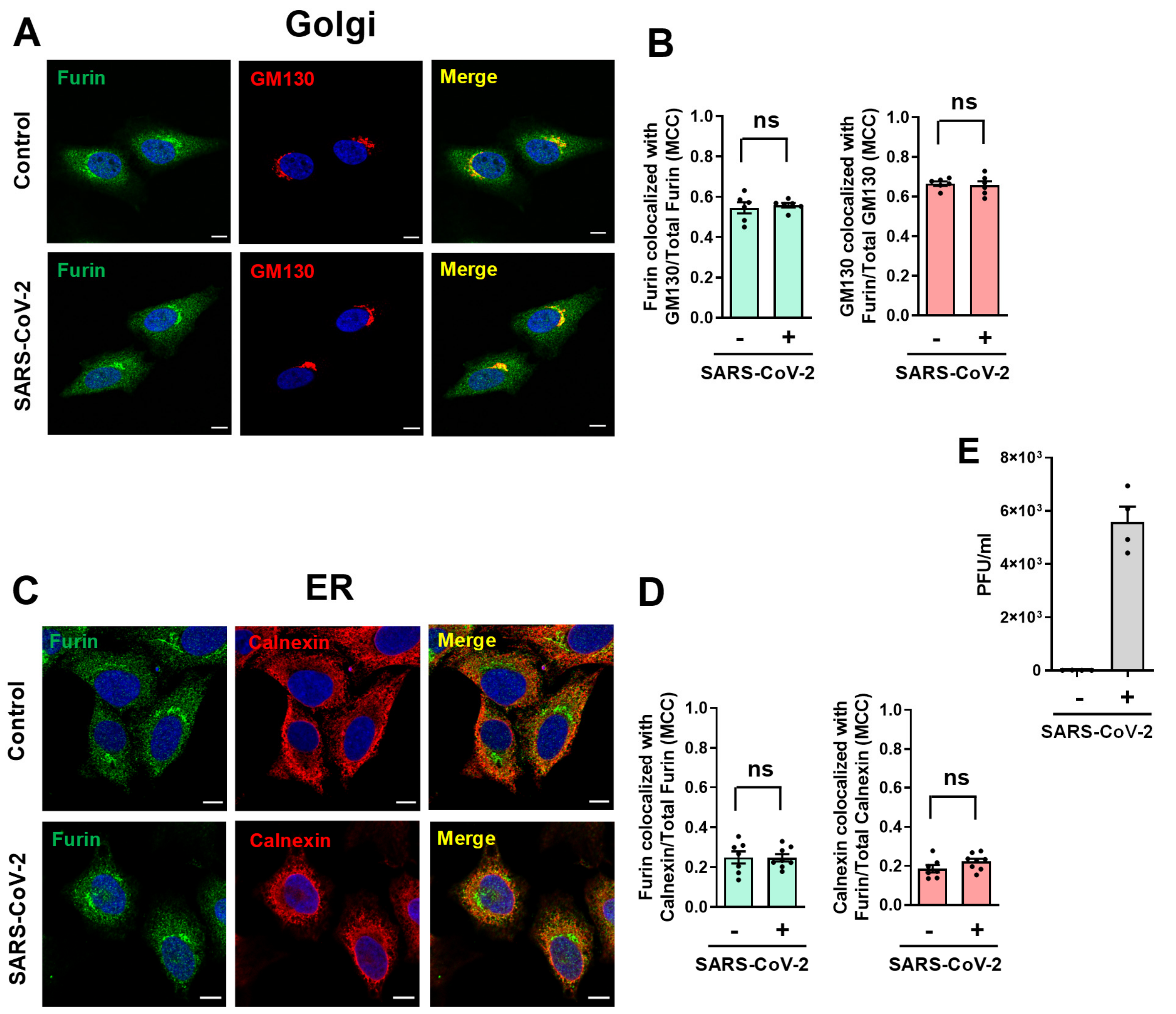


**Figure S9. SARS-CoV-2 infections do not affect cellular localization of furin.** The cellular localization of furin was analyzed using immunocytochemistry in HeLa cells stably expressing ACE2 (HeLa-ACE2) with or without SARS-CoV-2 infection. Furin was labelled with anti-Furin antibodies with green fluorophore-tagged antibodies. **(A and B)** The Golgi complex was labelled with anti-GM130 antibodies with red fluorophore-tagged antibodies after permeabilization. Representative immunofluorescence images are shown in (A). Quantification of colocalization between furin and GM130 using the Manders' colocalization coefficient (MCC) is summarized in (B, n = 6). **(C and D)** The ER was labelled with anti-calnexin antibodies with red fluorophore-tagged antibodies after permeabilization. Representative immunofluorescence images are shown in (C). Quantification of colocalization between furin and calnexin using MCC is summarized in (D, n = 7-8). The Golgi localization of furin is not significantly altered during SARS-CoV-2 infections. **(E)** The SARS-CoV-2 infections in HeLa-ACE2 cells were confirmed by quantifications of viral RNA in cell culture supernatant. Cells were infected with 0.01 MOI SARS-CoV-2, then culture supernatant was harvested at 24 h post-infection. The Ct values of quantitative PCR were converted into viral titer (pfu/mL). Bar graph data are shown as mean ± SEM. ns, not significant. Data were analyzed using a two-tailed Student's *t*-test (B and D).

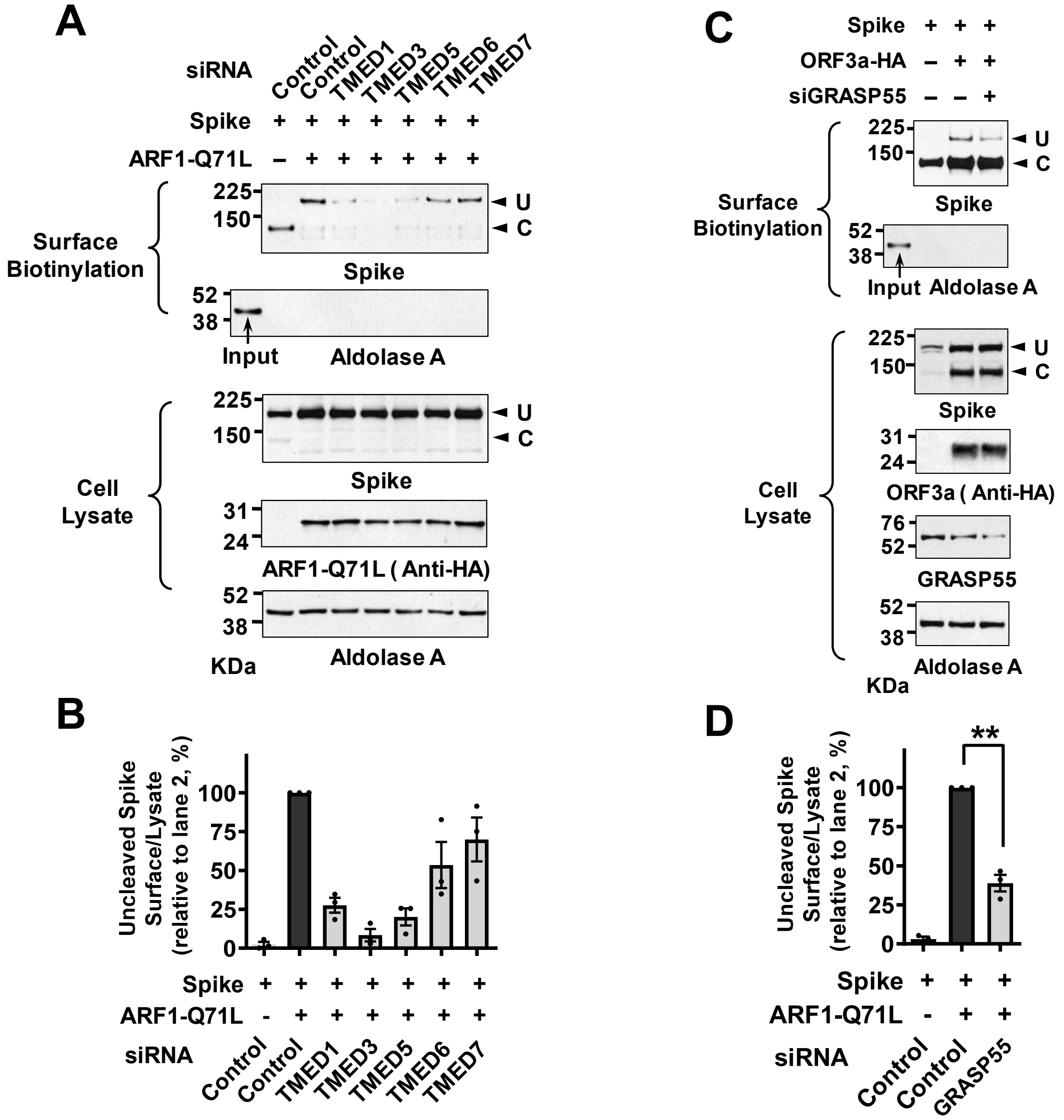


**Figure S10. TMEDγ proteins and GRASP55 are proteins involved in the UPS of SARS-CoV-2 Spike. (A and B)** Effects of individual TMEDγ (TMED1, 3, 5, 6, and 7) gene silencing (100 nM each, 48 h) on the ARF1-Q71L-induced UPS of SARS-CoV-2 Spike (S) were analyzed. The cell surface expression of uncleaved (U) and cleaved (C) S protein was analyzed using surface biotinylation assays in HEK293 cells. Plasmids encoding ARF1-Q71L were transfected to induce ER-to-Golgi blockade. Representative blots of surface biotinylation assays are shown in (A) and the results of multiple experiments are summarized in (B, n = 3). **(C and D)** Silencing of GRASP55 partially inhibits ORF3a-induced UPS of S. The effects of GRASP55 gene silencing (100 nM, 48 h) on the cell surface expression of S were analyzed with co-expression of SARS-CoV-2 ORF3a. Representative blots of surface biotinylation assays are shown in (C) and the results of multiple experiments are summarized in (D, n = 3). Bar graph data are shown as mean ± SEM. **P < 0.01. Data were analyzed using one-way analysis of variance, followed by Tukey's multiple comparison test.

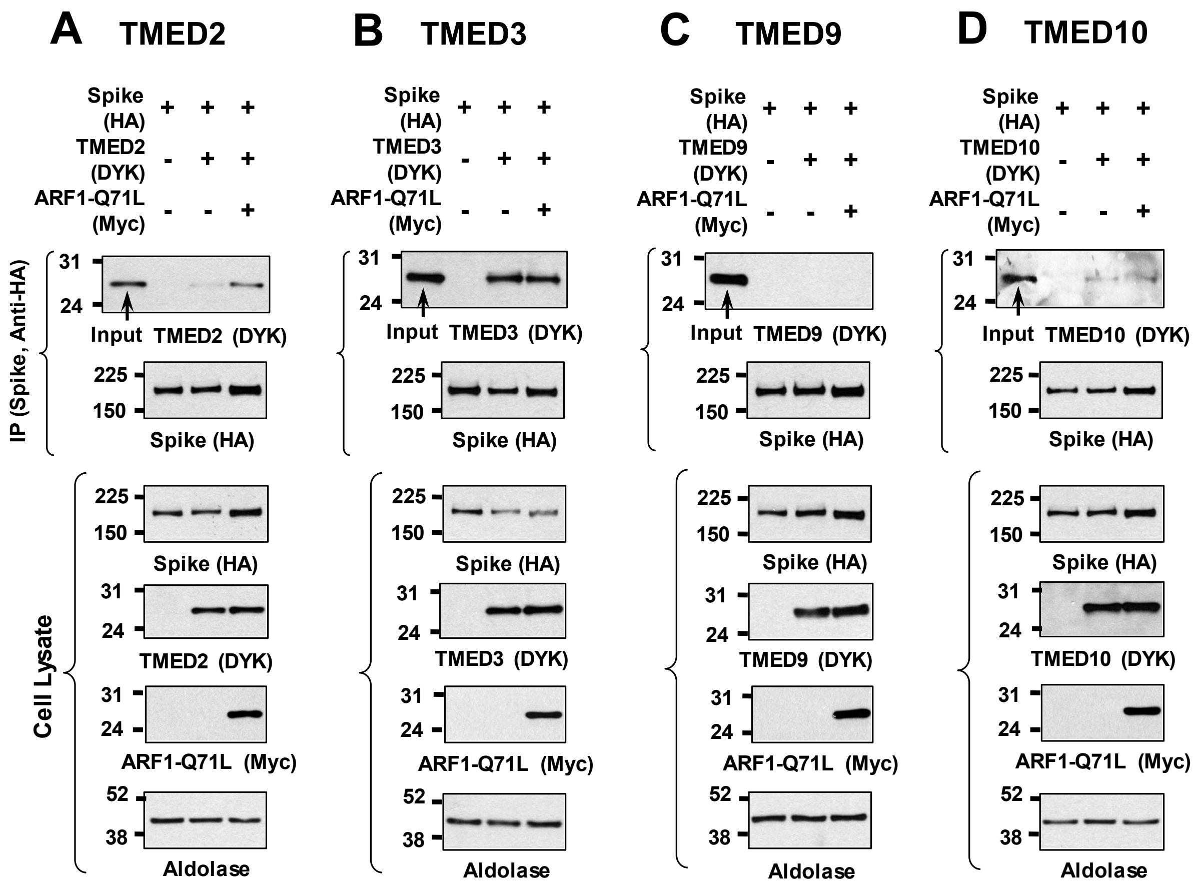


**Figure S11. TMED3 interacts with SARS-CoV-2 Spike.** Co-immunoprecipitation experiments with HA-tagged SARS-CoV-2 Spike and DYK-tagged TMED proteins [TMED2 (**A**), TMED3 (**B**), TMED9 (**C**), and TMED10 (**D**)] were performed in HEK293 cells. ARF1-Q71L was co-expressed in some cells to induce UPS. Protein samples were precipitated with anti-HA (Spike) antibodies and blotted with antibodies against each labeled protein. The Myc-tagged ARF1-Q71L was blotted with anti-Myc antibodies. SARS-CoV-2 spike most strongly binds to TMED3. Results shown are representative of three independent experiments.
